# Multi-Parameter Analysis of Photosynthetic and Molecular Responses in *Chlorella vulgaris* Exposed to Silver Nanoparticles and Ions

**DOI:** 10.3390/toxics13080627

**Published:** 2025-07-26

**Authors:** Bruno Komazec, Sandra Vitko, Biljana Balen, Mario Cindrić, Renata Biba, Petra Peharec Štefanić

**Affiliations:** 1 Department of Biology, Faculty of Science, University of Zagreb, Horvatovac 102a, HR-10000 Zagreb, Croatia; bruno.komazec@biol.pmf.unizg.hr (B.K.); sandra.vitko@biol.pmf.unizg.hr (S.V.); biljana.balen@biol.pmf.unizg.hr (B.B.); 2 Ruđer Bošković Institute, POB 1016, HR-10000 Zagreb, Croatia; mcindric@irb.hr (M.C.); rbiba@irb.hr (R.B.)

**Keywords:** silver nanoparticle coating, chlorophyll fluorescence, proteomics, 2-D electrophoresis, mass spectrometry, gene expression, qPCR

## Abstract

Due to widespread use of silver nanoparticles (AgNPs), the assessment of their potential harm to microalgal photosynthesis is crucial, as microalgae, together with cyanobacteria, contribute to approximately 50% of global oxygen production. This study investigated photosynthetic pigments, photosynthetic rate, chlorophyll *a* fluorescence, and the expression of photosynthesis-related genes and proteins in green alga *Chlorella vulgaris* after 72 h exposure to citrate- and cetyltrimethylammonium bromide (CTAB)-stabilized AgNPs, as well as silver ions (AgNO_3_), at concentrations allowing 75% cell survival (EC_25_). All treatments impaired photosynthetic performance. The most pronounced decreases in chlorophyll fluorescence parameters and photosynthetic rate, alongside elevated energy dissipation, were observed after exposure to AgNP-CTAB and AgNO_3_. AgNP-citrate had milder effects and induced compensatory responses, reflected in an increased performance index and upregulation of photosynthesis-related proteins. AgNP-CTAB induced the strongest downregulation of gene and protein expression, likely due to its higher EC_25_ concentration and cationic surface promoting interaction with photosynthetic structures. Although AgNO_3_ caused fewer molecular changes, it significantly disrupted photosynthetic function, suggesting a direct effect of Ag^+^ ions on photosynthesis-related proteins. Overall, the results highlight the role of AgNPs’ surface coatings and dosage in determining their phytotoxicity, with photosystem disruption and oxidative stress emerging as key mechanisms of action.

## 1. Introduction

Silver nanoparticles (AgNPs) are one of the most commonly used classes of nanomaterials. The global annual production of AgNPs is estimated to be around 500 tons, and the associated market value is estimated to increase from USD 1 billion in 2022 to USD 3 billion in 2024 [1]. Their broad-spectrum antimicrobial activity, particularly against antibiotic-resistant bacterial strains, underpins their wide application in biomedical fields. Moreover, AgNPs have demonstrated synergistic interactions with certain antibiotics, enhancing their therapeutic efficacy [2,3]. In addition to antimicrobial applications, AgNPs exhibit remarkable anticancer and antidiabetic properties and are currently being investigated for their potential in targeted drug delivery [4]. Due to their relatively low toxicity compared to conventional bactericidal agents, AgNPs are also used in a variety of commercial products, including textiles, disinfectants, and water purification systems [5].

A key property of AgNPs is their high reactivity, which results from their large surface-to-volume ratio. However, this reactivity also contributes to their physicochemical instability, which makes them susceptible to agglomeration, shape change, crystallization, and a variety of surface chemical reactions [6,7]. To increase the stability of AgNPs while maintaining their functional properties, surface modifications with steric or electrostatic stabilizers are commonly employed in their synthesis [8]. Steric stabilizers, such as polyvinylpyrrolidone (PVP) and polyethylene glycol (PEG), adsorb on the surface of the nanoparticles and form a physical barrier that prevents interactions between particles and aggregation [9]. In contrast, electrostatic stabilizers, such as citrate, cetyltrimethylammonium bromide (CTAB), sodium dodecyl sulphate (SDS), and polyethylenimine (PEI), impart a surface charge that causes electrostatic repulsion between the particles and thus increases dispersion stability [10,11]. The type of coating determines the surface charge of the nanoparticles, which can be either negative or positive; for example, citrate-functionalized AgNPs exhibit a negative surface charge, while CTAB-coated AgNPs acquire a positive charge. These surface charges not only prevent agglomeration but also modulate the interactions of AgNPs with biological systems and environmental matrices, which ultimately affects their bioavailability, reactivity, and potential toxicological effects [12].

The increasing use of AgNPs has led to their extensive release into various ecosystems [2,13], especially aquatic environments, where algae are often among the first organisms exposed to these nanomaterials [14,15]. Since algae, together with cyanobacteria, play a crucial role in global oxygen production through photosynthesis, their susceptibility to AgNP-induced toxicity poses a significant ecological problem [16,17]. The extent of AgNP toxicity to algal species is influenced by several physicochemical factors, including particle size, surface properties, type of surface coating, and the degree of Ag^+^ ion release due to nanoparticle dissolution [15]. Importantly, the mechanisms by which AgNPs and Ag^+^ ions exert toxic effects on algal cells differ significantly, and a comparative approach is essential to distinguish between nanoparticle-specific and ion-mediated responses. Ag^+^ ions are highly soluble, readily bioavailable, and capable of crossing cell membranes, where they disrupt enzymatic activity, impair membrane integrity, and interfere with electron transport and other essential cellular functions [11,18]. In contrast, AgNPs may exert toxicity through direct physical interactions with the cell wall or membrane, generation of reactive oxygen species (ROS) at the particle surface, or a gradual release of Ag^+^ ions after cellular uptake [8,12]. The rate and extent of Ag^+^ release from AgNPs are strongly influenced by surface coatings, with citrate-coated AgNPs typically showing higher dissolution and greater similarity to Ag^+^ behavior, while CTAB-coated AgNPs remain more stable and induce milder oxidative stress [8,11,13]. A recent study using *Chlorella vulgaris* has shown that AgNO_3_ (as a source of Ag^+^ ions) caused more acute oxidative damage than AgNPs, and that the toxicity of AgNPs was highly dependent on the surface stabilizer used, likely due to differences in their solubility and ion release [11,12]. Exposure to either AgNPs or Ag+ ions can induce the formation of ROS, which in turn damage important biomolecules such as lipids, proteins, and nucleic acids [19,20]. However, only Ag^+^ is readily taken up and accumulated intracellularly in green algae, where it can impair essential metabolic pathways and photosystem functions [11,12,18]. This includes disruption of thylakoid membranes and electron flow in photosystem II (PSII), resulting in diminished photosynthetic efficiency [21,22]. Indeed, studies have shown that exposure of *C. vulgaris* to uncoated AgNPs [23,24] and *Scenedesmus* to polyvinyl alcohol (PVA)-coated AgNPs [25] results in decreased chlorophyll content and structural disruption of PSII. Similarly, treatment of *Raphidocelis subcapitata* with PVP-coated AgNPs results in decreased photosynthetic performance [26].

Given the potential negative effects of AgNPs on algal photosynthesis, the present study investigated the impact of AgNPs stabilized with two different coatings, citrate (AgNP-citrate) and CTAB (AgNP-CTAB), on the photosynthetic performance of the green microalga *C. vulgaris*. The inclusion of AgNO_3_ treatments as a source of Ag^+^ ions was crucial to discriminating between ionic and nanoparticle-specific mechanisms of toxicity, as supported by previous green algal studies [8,11]. The use of AgNPs with different surface stabilizers allowed for the evaluation of whether nanoparticle surface chemistry differentially modulates photosynthetic responses in algal cells. Since AgNPs can interfere with pigment biosynthesis, photosynthetic efficiency, and related molecular pathways, and considering that surface coatings critically influence nanoparticle stability, surface charge, and reactivity, it is plausible that differently stabilized AgNPs elicit distinct physiological and molecular effects on algal photosynthesis. To assess these effects, we measured key photosynthetic parameters, including photosynthetic rate, chlorophyll and accessory pigment concentrations, and non-photochemical quenching (NPQ) capacity. Furthermore, to elucidate the underlying molecular mechanisms of algal responses, we conducted transcriptomic and proteomic analyses targeting genes and proteins associated with photosynthetic function. This integrative approach enabled a comprehensive assessment of both the physiological and molecular alterations induced by AgNP exposure.

## 2. Materials and Methods

### 2.1. Preparation and Physicochemical Characterization of AgNPs

Analytical-grade chemicals were used throughout the study and were purchased from Sigma-Aldrich (St. Louis, MO, USA), unless specified otherwise. For the synthesis of AgNPs, high-purity deionized water (Milli-Q system, 18.2 MΩcm^−1^, Merck Millipore, Billerica, MA, USA) was used.

The synthesis and physicochemical characterization of AgNP-citrate and AgNP-CTAB have already been described in detail in our earlier study [11]. Briefly, for the synthesis of AgNP-citrate, 120 mL of 0.017% (*w*/*v*) AgNO_3_ solution was prepared and heated to boiling. Then, 5 mL of 1% (*w*/*v*) sodium citrate solution was added with constant stirring. The reaction was monitored by the color change of the solution from transparent to pale yellow, after which the system was cooled to room temperature (RT) with a stream of cold water. AgNP-CTAB was prepared by mixing 60 mL of a cold 0.0167% (*w*/*v*) ascorbic acid solution with 65 mL of a cold aqueous solution containing 0.02 g AgNO_3_ and 0.0043 g CTAB. The addition was carried out slowly and in a controlled manner using a burette with constant stirring. The appearance of a light orange color indicated the formation of AgNPs.

A UV–vis spectrophotometer (Unicam, Cheshire, UK) was used to confirm the formation of AgNPs by detecting the characteristic surface plasmon resonance (SPR) peak. The hydrodynamic diameter (*d*_H_) and surface charge (ζ potential) of the nanoparticles were determined by dynamic light scattering (DLS) and electrophoretic light scattering (ELS), respectively, using a NanoBrook 90Plus (Brookhaven Instruments, Holtsville, NY, USA) equipped with a red laser light source (660 nm). Measurements were performed at RT, with light scattering detection for particle size performed at an angle of 180°, while an angle of 15° was used for ζ potential. The data obtained were analyzed using Zeta Plus software, version 5.71. The particle size results are presented as the average value of 10 consecutive measurements (mean ± standard error, *n* = 10), expressed as a volume distribution. The ζ potentials, on the other hand, represent the average of five measurements (*n* = 5), and the values were calculated on the basis of electrophoretic mobility using the Smoluchowski expression.

The amount of free Ag^+^ ions in both AgNP suspensions was determined using centrifugal ultrafiltration (Amicon Ultra-4 3K, Merck Millipore) through a membrane permeable to molecules smaller than 3 kDa, while the total silver concentration in the suspensions and filtrates was measured using inductively coupled plasma mass spectrometry (ICP-MS; ELAN DRC-e, Perkin Elmer, Waltham, MA, USA) after acidification with 10% nitric acid (HNO_3_). Quantification was based on a standard curve generated from known silver concentrations, while the limits of detection (LOD) and quantification (LOQ) were 0.2 mg kg^−1^ and 1 mg kg^−1^, respectively.

Visualization of AgNP-citrate and AgNP-CTAB suspensions was performed using transmission electron microscopy (TEM) using a TF20 instrument (FEI Tecnai G2, Hillsboro, OR, USA) equipped with an EDX detector for compositional analysis, as described in our previous study [11].

### 2.2. Culture Conditions

The culture of the alga *C. vulgaris* strain SAG/211-11b (Experimentelle Phykologie und Sammlung von Algenkulturen (EPSAG), Georg-August-Universität, Göttingen, Germany) was grown in 200 mL of liquid BBM medium [27] at a temperature of 24 °C under long-day conditions (16 h light, 8 h dark) with a light intensity of 80 µmol m^−2^ s^−1^ and constantly stirred with an orbital shaker during cultivation.

### 2.3. Determination of Cell Viability and EC_25_ Values

Since different surface coatings can have different phytotoxic effects, we used concentrations that allowed 75% cell survival (EC_25_) to enable a comparable assessment of their potential differential impact on photosynthesis. The analysis of cell survival of *C. vulgaris* after exposure to AgNPs and AgNO_3_ and the determination of EC_25_ concentrations were described in our previous study [11]. In brief, the algae were inoculated in BBM medium at an initial concentration of 1 × 10^5^ cells mL^−1^ and grown in a plant growth chamber for 4 days, followed by a 72 h treatment with different concentrations of AgNP-citrate, AgNP-CTAB, and AgNO_3_ according to OECD guidelines [28]. Cell viability was assessed using flow cytometry in combination with propidium iodide fluorescence staining, and EC_25_ values were determined using a four-parameter logistic regression model [29]. The EC_25_ concentrations obtained were 0.188 mg L^−1^ for AgNP-citrate, 0.895 mg L^−1^ for AgNP-CTAB, and 0.130 mg L^−1^ for AgNO_3_.

### 2.4. Assessment of AgNP Stability

The stability of AgNP-citrate, AgNP-CTAB, and AgNO_3_ in liquid BBM medium at concentrations corresponding to EC_25_ values has already been analyzed and described in detail in our previous work [11] using UV–vis spectroscopy and the DLS method, including ζ potential measurements. Briefly, changes in the position and intensity of the SPR peak, *d*_H_, and surface charge of the particles were monitored under the same conditions and at the same time points as the algal exposure experiment (1–72 h). Measurements were performed using a NanoBrook 90Plus particle size analyzer, and all samples were incubated under algal culture conditions. In short, the results showed that AgNP-citrate and AgNP-CTAB underwent initial agglomeration but exhibited relatively stable and predictable behavior over time, in contrast to AgNO_3_, which led to the in situ formation of AgNPs and showed greater variability in size and charge.

### 2.5. Silver Accumulation in Algal Cells

Determination of internalized silver in treated algal cells was previously reported in detail in our earlier study [11]. Briefly, the algal cells were centrifuged at 3500× *g* for 3 min at RT, and the resulting pellet was resuspended in 10 mL of buffer (2 mmol L^−1^ Na_2_HPO_4_ × 12H_2_O, 4 mmol L^−1^ NaH_2_PO_4_ × H_2_O, 9 mmol L^−1^ NaCl, 1 mmol L^−1^ KCl, pH 7.0). To remove silver from the cell surface, the suspension was incubated with 5 g of Amberlite HPR1100 ion-exchange resin (20–50 mesh, Sigma-Aldrich) and stirred at 4 °C for 2 h, followed by centrifugation at 4500× *g* for 30 min. The pellet was then washed with 1× PBS buffer (10 mmol L^−1^ Na_2_HPO_4_, 1.7 mmol L^−1^ KH_2_PO_4_, 2.7 mmol L^−1^ KCl, 130 mmol L^−1^ NaCl), centrifuged, and finally freeze-dried for 24 h. Subsequently, the cells were subjected to microwave digestion (ETHOS SEL Milestone, Shelton, CT, USA) according to a modified EPA 3051a method. In brief, the cells were first treated with 10 mL of concentrated HNO_3_ at 130 °C for 10 min, followed by 15 min at 180 °C. In the second digestion phase, the samples were treated with 1 mL of hydrogen peroxide at 85 °C for 5 min and at 130 °C for an additional 4 min. After cooling, the samples were diluted with 1% (*v*/*v*) HNO_3_ to a final volume of 50 mL. The silver content in the solutions was determined using an ELAN DRC-e ICP-MS instrument, calibrated using standard silver solutions of known concentrations. The limits of detection and quantification were 0.05 mg kg^−1^ and 0.1 mg kg^−1^, respectively, and the spike recovery test showed a recovery of 95.6%. In short, the results confirmed silver internalization in *C. vulgaris* cells for all treatments. The highest silver content was observed after AgNP-CTAB exposure (26.81 µg 10^−6^ cells), followed by AgNP-citrate (15.62 µg 10^−6^ cells), while the lowest accumulation occurred after AgNO_3_ treatment (9.96 µg 10^−6^ cells). Silver uptake correlated with the EC_25_ values of each treatment.

### 2.6. Quantification of Photosynthetic Pigment Concentrations

To determine the concentration of the photosynthetic pigments chlorophyll *a* (chl *a*), chlorophyll *b* (chl *b*), and carotenoids, the cell concentration in the suspensions was first measured. Based on these data, a volume of the cell suspension containing 7.5 × 10^6^ cells was aliquoted into tubes, whereupon the pigments were isolated according to a modified protocol described in [30]. The samples were centrifuged at 3500× *g* for 5 min at RT, and the supernatant was discarded. The pellet was then washed three times with ultrapure water, and centrifugation was repeated after each wash under identical conditions. The resulting pellet was then resuspended in cold 90% acetone and homogenized by adding silica beads (425–600 μm) at a frequency of 30 Hz for 4 min. After homogenization, the samples were centrifuged at 3500× *g* for 5 min and 4 °C, and the supernatant was collected in a dark tube and stored at 4 °C. The resuspension, homogenization, and centrifugation procedure were repeated two more times with 500 μL acetone each time, and all supernatants were combined and brought to a final volume of 2 mL with cold 90% acetone. The absorbance was measured spectrophotometrically at wavelengths of 470, 647, and 664 nm, and concentrations of chl *a*, chl *b*, and carotenoids were calculated according to the formulas of [31,32]. The results are expressed as μg chl *a* or *b* (10^6^ cells)^−1^ or μg carotenoids (10^6^ cells)^−1^ and represent the mean of 12 replicates ± standard error from two independent experiments.

### 2.7. Quantification of Photosynthetic Rate

The method of measuring oxygen release using the Chlorolab 2 device (Hansatech Instruments, King’s Lynn, UK) was applied to determine the rate of photosynthesis. To ensure consistency between treatments and controls, the cell concentration in the suspensions was first determined using an automatic cell counter (LUNA II, Logos Biosystems, Anyang, Republic of Korea). For the analysis, 1.5 mL of suspension with an equal number of algae cells was placed in the reaction chamber of the device. Measurements of oxygen release were conducted at a temperature of 30 °C with constant stirring at 30 rpm. The algal suspensions were sequentially illuminated with three different light intensities, 40, 80, and 120 μmol photons m^−2^ s^−1^, each applied for 20 min. After each illumination period, the suspensions were incubated in the dark for 20 min to determine the rate of cellular respiration. The values obtained are expressed as nmol of oxygen per hour per 10^6^ cells (nmol O_2_ h^−1^ (10^6^ cells)^−1^) and are presented as the mean of three replicates ± standard error based on three independent experiments.

### 2.8. Evaluation of Chlorophyll a Fluorescence Parameters

In vivo chlorophyll fluorescence parameters were analyzed using portable fluorometer AquaPen-C AP 110-C (Photon Systems Instruments, Drásov, Czech Republic), using the saturating pulse method. Prior to measurement, the algal samples were dark-adapted for 30 min. OJIP transients were recorded by applying a strong measuring pulse (~1590 μmol m^−2^ s^−1^, 455 nm), which induced a rapid fluorescence increase. Based on the recorded OJIP curves, selected parameters reflecting the efficiency of photosystem II were calculated: maximum quantum yield of PSII (*F_v_*/*F_m_*), performance index (*PI_abs_*), as well as absorption (*ABS*/*RC*), trapping (*TR*_0_/*RC*), electron transport (*ET*_0_/*RC*), and dissipation (*DI*_0_/*RC*) flux per reaction center (RC). All presented parameters are expressed as relative units (r.u.).

Additionally, non-photochemical quenching (*NPQ*) analysis was performed to estimate the capacity to dissipate excess absorbed energy via non-photochemical processes. After dark adaptation, the samples were exposed to continuous actinic light for 200 s with ten saturating pulses applied every 20 s, followed by a 390 s dark recovery phase, during which seven saturating pulses were applied every 60 s. From the data, the following parameters were calculated: effective quantum yield of photosystem II (*QY*), non-photochemical quenching (*NPQ*), and photochemical quenching (*Qp*). All presented parameters are expressed as relative units (r.u.).

### 2.9. Extraction of Total Soluble Proteins

For the analysis of differential protein expression, total soluble proteins were isolated using a modified phenol extraction method according to [33,34]. A cell suspension (400 mL in total) was first centrifuged at 3500× *g* for 5 min at RT. The cell pellet was then resuspended in 1.3 mL of extraction buffer consisting of 500 mmol L^−1^ Tris, 50 mmol L^−1^ EDTA, 700 mmol L^−1^ sucrose, 100 mmol L^−1^ KCl, 1 mmol L^−1^ phenylmethylsulfonyl fluoride (PMSF), and 2% (*v*/*v*) β-mercaptoethanol. The samples were homogenized using silica beads (425–600 μm) three times for 4 min at 30 Hz, with cooling between cycles using a Retsch homogenizer (MM200, Retsch, Haan, Germany). The homogenates were then incubated on ice in a shaker for 10 min. Each sample was then mixed with 2 mL phenol and incubated for 10 min at RT on a shaker. After incubation, the samples were centrifuged at 4500× *g* for 20 min at 4 °C, and the supernatants were mixed with 2 mL extraction buffer and incubated for 3 min at RT in a shaker. The samples were then centrifuged again at 4500× *g* for 20 min at 4 °C, and the supernatants were mixed with four volumes of cold precipitation solution (0.1 mol L^−1^ ammonium acetate in methanol). The prepared samples were incubated overnight at −20 °C. The resulting pellet was washed three times with precipitation solution and once with cold acetone. After each wash, the samples were centrifuged again under the same conditions for 10 min and dried in a desiccator. The dry pellet was resuspended in 400 μL isoelectric focusing (IEF) buffer [9 mol L^−1^ urea, 4% (*w*/*v*) CHAPS, 2 mg mL^−1^ DTT, 5.2 μL mL^−1^ ampholytes] and centrifuged at 20,000× *g* for 5 min at 4 °C. The concentration of protein was determined using a bovine serum albumin standard curve and a modified Bradford assay [33].

### 2.10. Two-Dimensional Electrophoresis

Prior to two-dimensional electrophoresis (2-DE), the protein extracts were diluted in IEF buffer to a final volume of 400 μL with 300 μg of protein per sample. To each sample, 5 μL of Coomassie Brilliant Blue (CBB) G-250 dye was added, and the samples were vortexed and centrifuged at 20,000× *g* for 5 min at RT. The supernatants were then added to the rehydration wells of IPG strips (Immobiline DryStrip, 13 cm, pH 3–10 NL, GE Healthcare, Chicago, IL, USA). Immobilines were covered with 900 μL of cover fluid (Immobiline DryStrip Cover Fluid, GE Healthcare) and rehydrated for 12–16 h at RT. After rehydration, the IPG strips were transferred to the Ettan IPGphor 3 system (GE Healthcare) for the initial phase of protein separation using the following IEF protocol: 500 V for 1 h (stepwise increase and hold), 1000 V for 1 h (gradient), 8000 V for 3 h (gradient), and 8000 V for 4 h (stepwise increase and hold), until a total of 30 kVh was reached. After electrophoresis, the IPG strips were stored at −80 °C.

After the first step of protein separation, the immobilized strips were incubated in equilibration buffer (0.05 mol L^−1^ Tris-HCl, 6 mol L^−1^ urea, 2% (*w*/*v*) SDS, pH 8.8) with 130 mmol L^−1^ DTT (reduction step) for 15 min and then in the same buffer with 135 mmol L^−1^ iodoacetamide (alkylation step) for 15 min. After rinsing in electrode buffer, the strips were placed on 12% SDS-polyacrylamide gels, while Whatman paper soaked with 5 μL of molecular weight markers was placed on the opposite side of the gel. SDS-PAGE was performed in a PROTEAN II xi Cell system (BioRad, Hercules, CA, USA) at 100 V for the first 30 min and then at 220 V until completion, with the temperature kept constant by water cooling.

### 2.11. Gel Imaging and Data Processing

To analyze the effect of AgNPs or AgNO_3_ on differential protein expression, the entire experiment was repeated three times. After 2-DE, the gels were stained with CBB R-250 (0.1% (*w*/*v*) CBB R-250, 45% (*v*/*v*) ethanol, 10% (*v*/*v*) glacial acetic acid) for 60 min at RT and then destained with a solution of 10% acetic acid and 20% (*v*/*v*) methanol for 60 min at RT and overnight at 4 °C. The gels were scanned using an Epson Perfection V700 scanner (Epson, Nagano, Japan), and the protein spots were analyzed using ImageMaster 2D Platinum 7.0 (GE HealthCare, Chicago, IL, USA). Spots with expression changes of at least 1.5-fold compared to the control were selected for further analysis.

### 2.12. In-Gel Digestion and Peptide Purification

Selected protein spots were excised from the 2-D gels with a cut pipette tip and incubated in 10% (*v*/*v*) acetic acid and 40% (*v*/*v*) methanol at RT with shaking at 550 rpm until completely destained. The gel pieces were then washed three times for 30 min in 5 mmol L^−1^ ammonium bicarbonate (ABC) and 50% (*v*/*v*) acetonitrile (ACN) at RT with shaking at 900 rpm, dehydrated in 100% ACN (20 min), and reduced for 45 min in 10 mmol L^−1^ DTT prepared in 20 mmol L^−1^ ABC at 56 °C and 900 rpm. Alkylation was performed by incubation in the dark in 55 mmol L^−1^ iodoacetamide in 20 mmol L^−1^ ABC for 30 min, followed by two 20 min washes in 5 mmol L^−1^ ABC and 50% (*v*/*v*) ACN and re-dehydration in 100% ACN. The gels were dried in a vacuum centrifuge (Concentrator plus, Eppendorf, Thermo Scientific, Waltham, MA, USA) at 30 °C and then incubated overnight in 50 µL 20 µg mL^−1^ trypsin prepared in 20 mmol L^−1^ ABC at 37 °C and 600 rpm. After digestion, the supernatants were transferred to clean tubes, and the gels were incubated twice for 30 min in 50 µL of 50% (*v*/*v*) ACN and 1% (*v*/*v*) trifluoroacetic acid (TFA), then for an additional 30 min in 80% (*v*/*v*) ACN and 1% (*v*/*v*) TFA at RT and 900 rpm. All supernatants were pooled and dried in a vacuum centrifuge at 30 °C to a volume of approximately 50 µL.

The resulting peptides were purified by reverse-phase solid-phase microextraction (SPME) using a C18 stationary phase (Agilent, Santa Clara, CA, USA) and an AssayMap automated liquid handling platform (Agilent, Santa Clara, CA, USA). The stationary phase was activated with 100 µL methanol and equilibrated with 100 µL 2% (*v*/*v*) ACN and 1% (*v*/*v*) formic acid (FA). Subsequently, 40 µL of peptide solution was loaded onto the stationary phase at a flow rate of 10 µL min^−1^ and washed with 100 µL of 0.1% (*v*/*v*) FA. The peptides were eluted in 25 µL 80% (*v*/*v*) ACN and 0.1% (*v*/*v*) FA, dried in a vacuum centrifuge, and stored at −80 °C.

### 2.13. Qualitative Proteomics Analysis

Qualitative protein analysis was performed using liquid chromatography coupled with quadrupole time-of-flight mass spectrometry (LC-QTOF-MS). The peptides obtained were dissolved in 10 µL of 0.1% (*v*/*v*) FA and transferred to a glass vial. Eight microliters were injected per analysis, and data acquisition was performed in Auto MS/MS mode with positive ESI ionization, using an Agilent 6545 Q-TOF mass spectrometer (Agilent, Santa Clara, CA, USA) coupled to an Agilent 1290 Infinity II UHPLC system. MassHunter 10.0 software (Agilent, Santa Clara, CA, USA) was used for instrument control, data acquisition, and spectral evaluation. Peptides were separated on an AdvanceBio Peptide Map analytical column (1.0 × 150 mm, 2.7 µm, Agilent), and the analysis was conducted under defined UHPLC (Table S1) and MS (Table S2) conditions. Data were processed using ProteinPilot 4.5 software (AB Sciex, Darmstadt, Germany). The false discovery rate (FDR) was set at ˂ 1. The identified proteins were characterized by searching the UniProt database Taxon ID 3077 *C. vulgaris* and using the ProtParam tool (Expasy, Swiss Institute of Bioinformatics, Geneva, Switzerland).

### 2.14. Quantification of Chloroplast-Encoded Genes by qPCR

Total RNA was extracted from *C*. *vulgaris* cells by centrifugation of 200 mL of cell suspension at 3500× *g* for 5 min at RT. After centrifugation, the pellet was washed three times with ultrapure water, with centrifugation under the same conditions after each wash. After the last wash, the pellet was resuspended in lysis buffer from the Quick-RNA™ Miniprep Plus Kit (Zymo Research, Irvine, CA, USA), and silica beads were added. Cell disruption was performed using a Retsch MM200 homogenizer (Retsch, Germany) at a frequency of 30 Hz for 12 min. Subsequent RNA isolation steps were performed according to the manufacturer’s instructions.

RNA concentration and purity were determined using a NanoDrop™ 1000 spectrophotometer (Thermo Fisher Scientific, Waltham, MA, USA). For cDNA synthesis, 1 µg of total RNA was used in a 20 µL reaction containing 10 µL of 2× ZymoScript™ RT PreMix (ZymoScript™ RT PreMix Kit, Zymo Research), the appropriate volume of RNA, and nuclease-free water. Reverse transcription was performed under the following thermal conditions: 25 °C for 2 min, 55 °C for 10 min, 42 °C for 30 min, and 95 °C for 1 min. The obtained cDNA was diluted with nuclease-free water to achieve a final concentration of 10 ng µL^−1^ for downstream applications.

Quantitative PCR (qPCR) was performed in a total volume of 15 µL, containing 2 µL of diluted cDNA, 1× GoTaq® qPCR Master Mix (Promega, Madison, WI, USA), and 200 nmol L^−1^ of each gene-specific primer (Table 1). Reactions were performed on a Magnetic Induction Cycler (Mic qPCR, Bio Molecular Systems, Queensland, Australia) using the following thermal cycling program: 95 °C for 5 min, followed by 40 cycles of 95 °C for 5 s and 60 °C for 30 s. The *CYP* gene (Table 1) was used as an internal reference for normalization [35]. Relative gene expression levels were calculated using the ΔΔC_t_ method [36,37] and normalized to the expression levels observed in the control culture.

### 2.15. Statistical Analysis

Before the statistical analysis, outliers were identified using Tukey’s fence method (k = 1.5). Normality and homogeneity of variances were tested using the Shapiro–Wilk and Levene’s tests, respectively. As assumptions were met (*p* > 0.05), parametric tests were applied. Differences in pigment content, photosynthetic rate, and chl *a* fluorescence parameters were analyzed using one-way ANOVA followed by the Newman–Keuls post hoc test. Gene expression data were analyzed using one-way ANOVA followed by Tukey’s HSD post hoc test. Statistical significance was set at *p* ≤ 0.05, and all analyses were performed using STATISTICA 14.0.0.15 (TIBCO Software Inc., Palo Alto, CA, USA).

## 3. Results

### 3.1. Physicochemical Characterization of AgNPs

Stock suspensions of synthesized AgNP-citrate and AgNP-CTAB were characterized using a combination of UV-Vis spectroscopy, TEM, DLS, and ELS. The UV-Vis spectra revealed SPR peaks at 420 nm for AgNP-citrate and at 455 nm for AgNP-CTAB, confirming successful synthesis (Figure S1A,E). DLS analysis indicated average *d*_H_ of 43.1 ± 0.8 nm for AgNP-citrate and 62.2 ± 1.5 nm for AgNP-CTAB (Table S3). TEM imaging showed that most particles were spherical, with a small proportion exhibiting a rod-like morphology (Figure S1B,F). The particle diameters observed in TEM ranged between 40 and 60 nm for AgNP-citrate and 50–70 nm for AgNP-CTAB. Elemental mapping and EDX analysis verified the presence of silver in the nanoparticles (Figure S1C,D,G,H). The measured ζ potentials was −45.62 ± 2.68 mV for AgNP-citrate and 39.89 ± 1.79 mV for AgNP-CTAB. Total silver concentrations, as well as the proportion of dissolved ionic silver (Ag^+^), were determined by ICP-MS and are presented in Table S3.

### 3.2. Quantity of Photosynthetic Pigments

**Figure 1:**
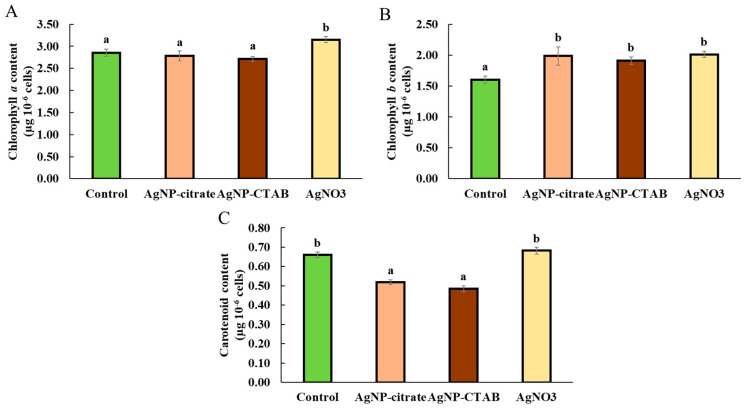
Content of chlorophyll *a* (**A**), chlorophyll *b* (**B**), and carotenoids (**C**) in *C. vulgaris* control cells and cells exposed to AgNP-citrate (0.188 mg L^−1^), AgNP-CTAB (0.895 mg L^−1^), and AgNO_3_ (0.130 mg L^−1^) for 72 h. Values represent mean ± standard error from two independent experiments, each with 6 replicates (n = 12). Treatments that differ significantly at *p* ≤ 0.05 (one-way ANOVA followed by Newman–Keuls post hoc test) are labeled with different letters.

Only exposure to AgNO_3_ resulted in a significant increase in chl *a* content (Figure 1A), while all treatments significantly elevated chl *b* content, with a similar increase compared to the control (Figure 1B). Interestingly, treatments with both types of AgNPs significantly and equally decreased the carotenoid content compared to the control, while treatment with AgNO_3_ had no effect (Figure 1C).

### 3.3. Rate of Photosynthesis

**Figure 2:**
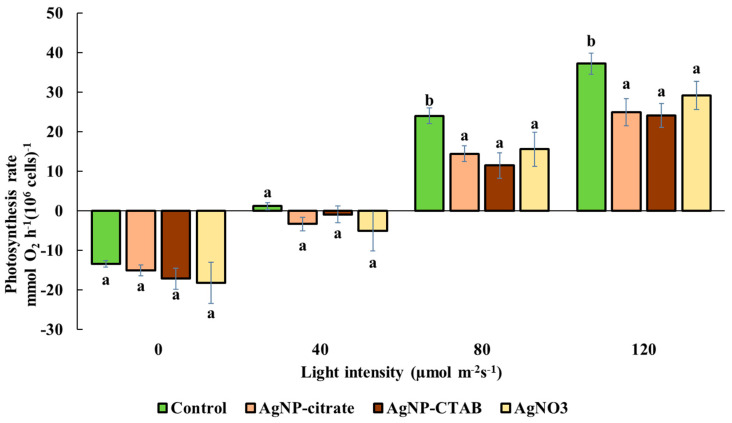
Photosynthetic rate in *C. vulgaris* control cells and cells exposed to AgNP-citrate (0.188 mg L^−1^), AgNP-CTAB (0.895 mg L^−1^), and AgNO_3_ (0.130 mg L^−1^) for 72 h at increasing light intensities (0, 40, 80, and 120 µmol photons m^−2^ s^−1^). Values represent mean ± standard error from three replicates. Treatments that differ significantly at *p* ≤ 0.05 (one-way ANOVA followed by Newman–Keuls post hoc test) are labeled with different letters.

None of the treatments resulted in a significant change in the amount of oxygen produced under dark conditions (0 µmol photons m^−2^ s^−1^) or under illumination at an intensity of 40 µmol photons m^−2^ s^−1^, although a slight decrease in the measured oxygen levels was observed for in treatments compared to the control. At light intensities of 80 and 120 µmol photons m^−2^ s^−1^, a significantly reduced photosynthetic rate with a similar value was recorded after exposure to all treatments compared to the control (Figure 2).

### 3.4. Chlorophyll a Fluorescence

**Figure 3:**
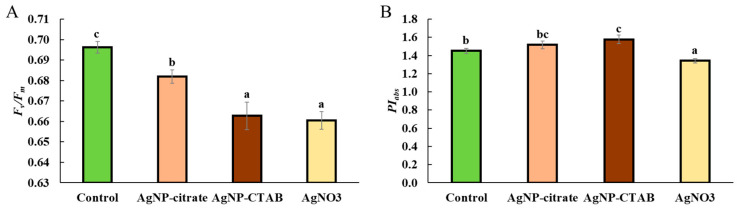
Maximum quantum yield of PSII (*F_v_*/*F_m_*) (**A**) and performance index (*PI_abs_*) (**B**) in *C. vulgaris* control cells and cells exposed to AgNP-citrate (0.188 mg L^−1^), AgNP-CTAB (0.895 mg L^−1^), and AgNO_3_ (0.130 mg L^−1^) for 72 h. Values represent mean ± standard error from two independent experiments, with 6 replicates each (n = 12). Treatments that differ significantly at *p* ≤ 0.05 (one-way ANOVA followed by Newman–Keuls post hoc test) are labeled with different letters.

All treatments significantly reduced the maximum quantum yield of PSII (*F_v_*/*F_m_*) compared to the control, with the strongest effect observed after treatment with AgNP-CTAB and AgNO_3_ (Figure 3A). On the other hand, the *PI_abs_* value (performance index) significantly decreased after treatment with AgNO_3_, while it increased following treatment with both types of AgNPs compared to the control (Figure 3B).

**Figure 4:**
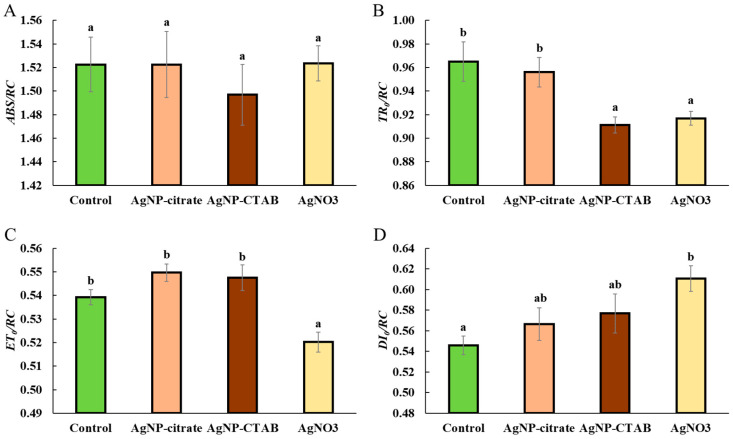
Absorption flux per reaction center (*ABS*/*RC*) (**A**), trapping flux per reaction center (*TR*_0_/*RC*) (**B**), electron transport flux per reaction center (*ET*_0_/*RC*) (**C**), and dissipation flux per reaction center (*DI*_0_/*RC*) (**D**) in *C. vulgaris* control cells and cells exposed to AgNP-citrate (0.188 mg L^−1^), AgNP-CTAB (0.895 mg L^−1^), and AgNO_3_ (0.130 mg L^−1^) for 72 h. Values represent mean ± standard error from two independent experiments, with 6 replicates each (n = 12). Treatments that differ significantly at *p* ≤ 0.05 (one-way ANOVA followed by Newman–Keuls post hoc test) are labeled with different letters.

None of the treatments caused a significant change in the absorption flux per RC (*ABS*/*RC*) compared to the control (Figure 4A), although exposure to AgNP-CTAB resulted in a slight decrease. The trapping flux per RC (*TR*_0_/*RC*) remained unchanged after treatment with AgNP-citrate, while AgNP-CTAB and AgNO_3_ treatments significantly and similarly reduced this value compared to both the control and AgNP-citrate treatment (Figure 4B). The amount of electron transport flux per RC (*ET*_0_/*RC*) increased slightly after exposure to AgNP-citrate and AgNP-CTAB compared to the control, although this increase was not statistically significant. In contrast, AgNO_3_ treatment significantly reduced *ET*_0_/*RC* compared to the control and the other treatments (Figure 4C). The dissipation flux per RC (*DI*_0_/*RC*) increased slightly after treatment with both types of AgNPs, although this increase was not statistically significant, while exposure to AgNO_3_ resulted in a significant increase in *DI*_0_/*RC* value compared to the control (Figure 4D).

**Figure 5:**
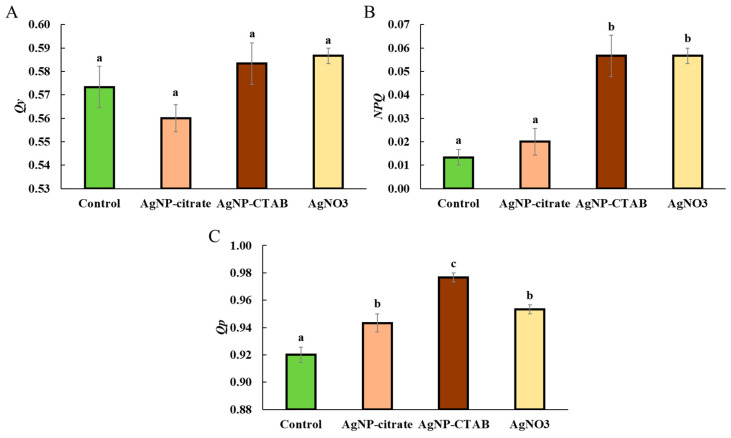
Effective quantum yield of PSII (*QY*) (**A**), non-photochemical quenching (*NPQ*) (**B**), and photochemical quenching coefficient (*Qp*) (**C**) in *C. vulgaris* control cells and cells exposed to AgNP-citrate (0.188 mg L^−1^), AgNP-CTAB (0.895 mg L^−1^), and AgNO_3_ (0.130 mg L^−1^) for 72 h. Values represent mean ± standard error from 6 replicates. Treatments that differ significantly at *p* ≤ 0.05 (one-way ANOVA followed by Newman–Keuls post hoc test) are labeled with different letters.

All treatments had distinct effects on the non-photochemical quenching (NPQ) parameters of chl *a* in *C. vulgaris* (Figure 5). The effective quantum yield of PSII (QY) remained unchanged following all treatments, with no statistically significant differences observed when compared to the control (Figure 5A). Furthermore, the NPQ value slightly increased after exposure to AgNP-citrate, although this increase was not statistically significant. A significant increase in NPQ compared to the control was observed only after treatment with AgNP-CTAB and AgNO_3_ (Figure 5B). A similar pattern was observed for the photochemical quenching coefficient (Qp), which increased following all treatments. Treatments with AgNP-citrate and AgNO_3_ caused a similar increase in Qp relative to the control, while the most pronounced increase was recorded after exposure to AgNP-CTAB (Figure 5C).

### 3.5. Protein Expression Profiling in C. vulgaris Using 2-DE

**Figure 6:**
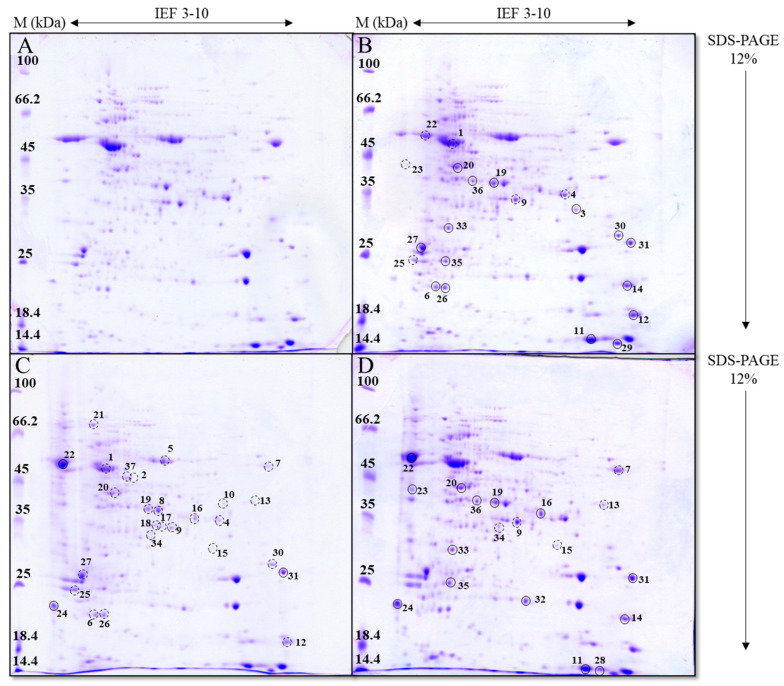
Total soluble proteins in *C. vulgaris* control cells (**A**) and cells exposed to AgNP-citrate (0.188 mg L^−1^) (**B**), AgNP-CTAB (0.895 mg L^−1^) (**C**), and AgNO_3_ (0.130 mg L^−1^) (**D**) for 72 h, separated by 2-DE. M—molecular weight marker (kDa), IEF 3–10—relative protein position in the gel according to their isoelectric point, SDS-PAGE—relative protein position in the gel according to their molecular weight. Proteins with expression levels significantly different from the control (*p* ≤ 0.05) are labeled with the numbers 1–37. Proteins with increased expression compared to the control are marked with filled circles (○), while those with decreased expression are marked with dashed circles (◌).

For each treatment, three 2-D gels were prepared from three independent experiments, and representative gels of the control and treated cells (AgNP-citrate, AgNP-CTAB, and AgNO_3_) are shown in Figure 6. Protein spots exhibiting a change in abundance of at least 1.5-fold relative to the control were selected for identification using mass spectrometry.

After treatment with both types of AgNPs and AgNO_3_, a total of 37 protein spots with different expressions compared to the control were detected in *C. vulgaris* cells and identified by mass spectrometry (Figure 6). The lowest number of proteins (20) with altered expression was detected after AgNO_3_ treatment, of which 14 proteins were upregulated and five proteins were downregulated (Figure 6D). Exposure to AgNP-citrate led to the identification of 21 differentially expressed proteins, of which 15 showed increased expression and 6 showed decreased expression (Figure 6B). The highest number of differentially expressed proteins (27) was found after exposure to AgNP-CTAB, with 24 proteins upregulated and only three proteins downregulated (Figure 6C).

All 37 differentially expressed proteins were identified by a database search (Table 2), of which five proteins showed expression changes after all treatments. Eight proteins were differentially expressed only after treatment with AgNPs, with AgNP-citrate stimulating their expression, while AgNP-CTAB mostly resulted in decreased expression. Six proteins were differentially expressed after exposure to AgNP-citrate and AgNO_3_, with the majority showing downregulation after both treatments. Similarly, six proteins showed altered, mostly decreased, expression after treatment with AgNP-CTAB and AgNO_3_. Furthermore, two proteins were upregulated only after treatment with AgNP-citrate or AgNO_3_, while eight proteins showed decreased expression only after treatment with AgNP-CTAB.

Using the UniProt database and the ProtParam tool, data on the molecular weight, isoelectric point, cellular localization, molecular function, and biological processes of the identified proteins were obtained and used for their categorization. These data are presented in Table S4.

### 3.6. Functional Categorization and Expression Patterns in C. vulgaris

**Figure 7:**
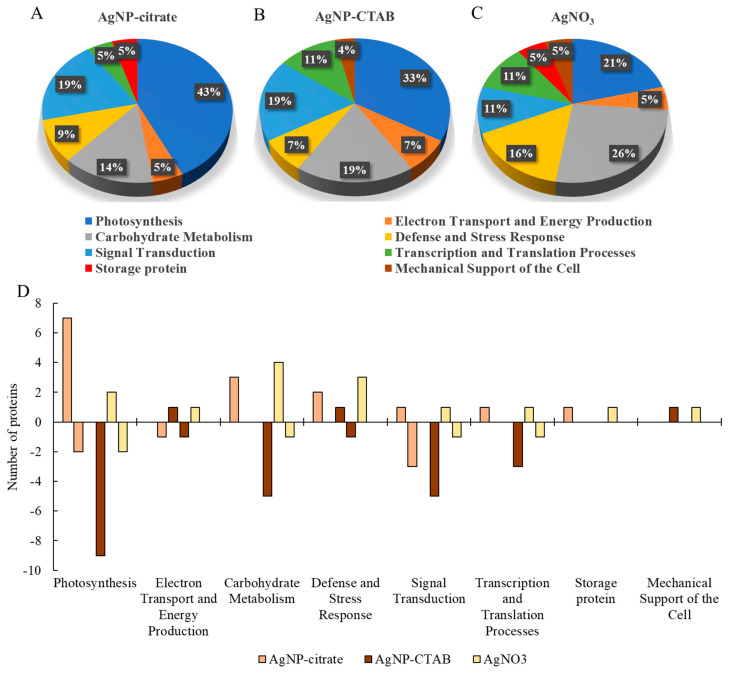
Distribution of functional categories of differentially expressed proteins in *C. vulgaris* cells exposed to AgNP-citrate (0.188 mg L^−1^) (**A**), AgNP-CTAB (0.895 mg L^−1^) (**B**), and AgNO_3_ (0.130 mg L^−1^) (**C**) for 72 h, and the number of proteins within each functional category showing increased (positive y-axis) or decreased (negative y-axis) expression (**D**) compared to the control.

Differentially expressed proteins were classified into eight categories based on their general functions: Photosynthesis, Electron Transport and Energy Production, Carbohydrate Metabolism, Defense and Stress Response, Signal Transduction, Transcription and Translation Processes, Storage Proteins, and Mechanical Support of the Cell (Figure 7).

After treatment of *C. vulgaris* with AgNP-citrate, AgNP-CTAB, and AgNO_3_, the majority of differentially expressed proteins were associated with the Photosynthesis and Carbohydrate Metabolism categories, while other categories were represented to a lesser extent (Figure 7A–C).

Proteins associated with the Photosynthesis category were most abundant after treatment with both AgNP types (43% and 33% of all differentially expressed proteins for AgNP-citrate and AgNP-CTAB, respectively), and proteins of this category were also significantly represented after treatment with AgNO_3_ (21% of all differentially expressed proteins). Among these proteins, those related to PSI and PSII and the RuBisCo subunits were most frequently differentially expressed. After treatment with AgNP-citrate, most proteins related to photosynthesis showed decreased expression, with exceptions such as increased expression of the RuBisCo small subunit and proteins associated with PSI (e.g., PSI subunit PsaD) and PSII (e.g., D1 protein) (Figure 7D). After exposure to AgNP-CTAB, all photosynthesis-related proteins were downregulated, including the large subunit of RuBisCo and beta carbonic anhydrase. After AgNO_3_ treatment, decreased expression of the chlorophyll-binding proteins of PSI and PSII was observed, while the RuBisCo small subunit was upregulated.

Proteins associated with the Carbohydrate Metabolism category were equally represented after treatments with AgNO_3_ (five proteins; 26% of all differentially expressed proteins) and AgNP-CTAB (five proteins; 19% of all differentially expressed proteins), while exposure to AgNP-citrate resulted in increased expression of three proteins (14% of all differentially expressed proteins). Proteins involved in glycolysis, the citric acid cycle, and the degradation of small molecules were predominantly upregulated after exposure to AgNO_3_ and AgNP-citrate, while treatment with AgNP-CTAB generally led to their downregulation.

The Defense and Stress Response category was represented by four proteins, three of which showed increased expression after treatment with AgNO_3_, while treatments with AgNPs led to changes in the expression of two proteins. The protein Hsp70B was differentially expressed and downregulated only after exposure to AgNP-CTAB, while the antifreeze protein exhibited increased expression in all treatments.

Proteins belonging to the categories of Signal Transduction and Transcription and Translation Processes were detected in all treatments. However, the highest number in both categories was recorded after exposure to AgNP-CTAB, and their expression decreased. While some protein kinases and rhodanese were downregulated after AgNP-citrate treatment, AgNO_3_ treatment resulted in increased expression of proteins involved in sulphur transport and RNA recognition motif-containing proteins.

The Electron Transport and Energy Production category was represented by only two proteins, of which the ATP synthase gamma chain was only detected with reduced expression upon AgNP-CTAB treatment. In contrast, the beta subunit of ATP synthase was detected in all treatments, but its expression varied depending on the treatment, being downregulated after exposure to AgNP-citrate and upregulated after exposure to AgNP-CTAB and AgNO_3_.

The categories of the Storage Proteins and the Mechanical Support of the Cell were each represented by only one protein and thus accounted for the smallest proportion of differentially expressed proteins. Both proteins were upregulated, although not by the same treatments; the storage protein Cupin type 1 showed increased expression after exposure to AgNP-citrate and AgNO_3_, while cytoskeletal protein actin was upregulated with AgNP-CTAB and AgNO_3_.

### 3.7. Effect of Silver Nanoparticles and Ions on Chloroplast-Encoded Genes

**Figure 8:**
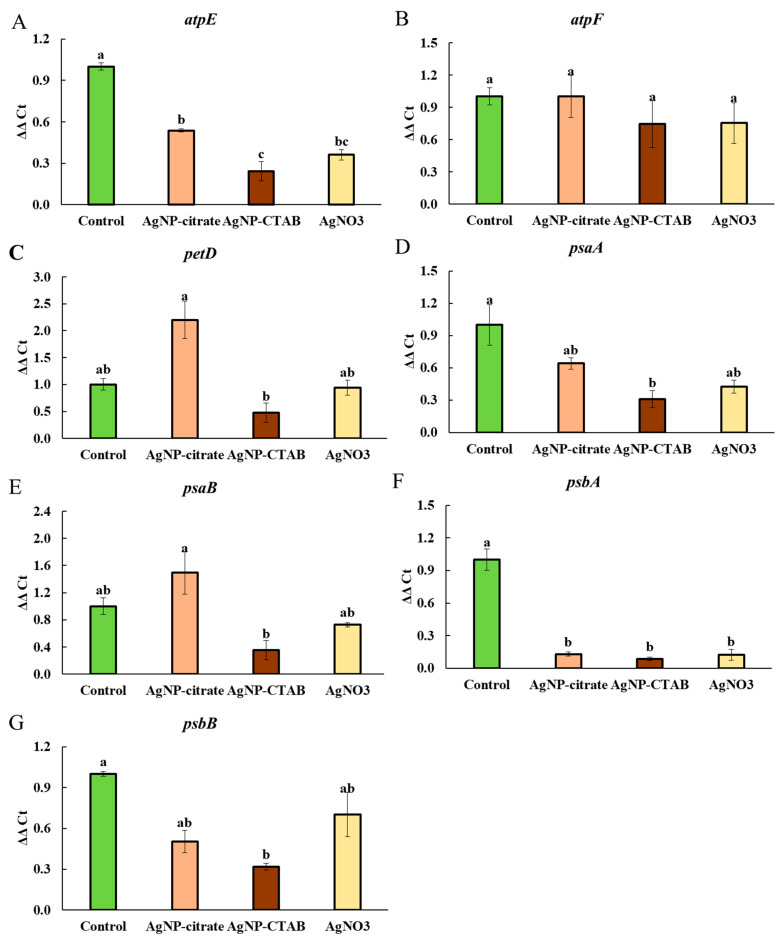
Relative gene expression (ΔΔC_t_) of (**A**) *atpE*, (**B**) *atpF*, (**C**) *petD*, (**D**) *psaA*, (**E**) *psaB*, (**F**) *psbA*, and (**G**) *psbB* in *C. vulgaris* control cells and cells exposed to AgNP-citrate (0.188 mg L^−1^), AgNP-CTAB (0.895 mg L^−1^), and AgNO_3_ (0.130 mg L^−1^) for 72 h. Values represent relative mean ± standard error from three replicates normalized to control (set as 1). Treatments that differ significantly at *p* ≤ 0.05 (one-way ANOVA followed by Tukey’s HSD post hoc test) are labeled with different letters.

To evaluate the effects of AgNPs and Ag^+^ ions on the expression of photosynthesis-related genes in *C. vulgaris*, we measured the transcript levels of seven chloroplast-encoded genes (*atpE*, *atpF*, *petD*, *psaA*, *psaB*, *psbA*, and *psbB*) in control cells and those exposed to EC_25_ concentrations of AgNP-citrate, AgNP-CTAB, or AgNO_3_. These genes were selected because they encode key subunits of photosynthetic protein complexes such as ATP synthase (*atpE*, *atpF*), cytochrome b6f complex (*petD*), Photosystem I (*psaA*, *psaB*), and Photosystem II (*psbA*, *psbB*), which were found to be differentially expressed at the protein level in our proteomic analysis. Gene expression analysis revealed significant variations between the different treatments (Figure 8). The expression of *atpE* was significantly reduced in all treatments compared to the control (Figure 8A), with the lowest expression observed upon exposure to AgNP-CTAB, followed by AgNO_3_ and AgNP-citrate. A similar pattern was observed for *psaA* (Figure 8D) and *psbB* (Figure 8G), although significantly reduced values were only recorded upon exposure to AgNP-CTAB compared to the control. Expression of *psbA* was significantly downregulated in all treatments compared to the control, with no significant differences between them (Figure 8F). The transcript levels of *petD* and *psaB* significantly decreased in cells after treatment with AgNP-CTAB than in cells after treatment with AgNP-citrate (Figure 8C,E), while control and AgNO_3_ treatment resulted in mean expression levels that were not significantly different from those of AgNP treatments. Interestingly, the expression of *atpF* remained stable in all treatments, with no significant differences compared to the control, indicating that this gene was stably expressed under the tested conditions (Figure 8B).

## 4. Discussion

Through photosynthesis, cyanobacteria and algae produce half of all atmospheric oxygen [16,38], and the process itself depends on the light energy absorbed by pigments, of which chl *a* and *b* and carotenoids are the most important [39]. Therefore, the composition and concentration of photosynthetic pigments have a significant impact on the amount of light absorbed and, consequently, on the overall efficiency of photosynthesis in algae [40]. It is known from the literature that algae can increase the content of photosynthetic pigments, especially chl *a* and *b*, under stress conditions such as temperature changes and elevated salinity, as well as after exposure to metals and metal nanoparticles, to increase photosynthetic activity and energy production [41,42]. In this study, the effect of the investigated treatments on the content of the analyzed photosynthetic pigments was quite different: the content of chl *a* increased only by exposure to AgNO_3_, while the content of chl *b* was affected by both AgNO_3_ and AgNPs. On the contrary, treatments with both types of AgNPs decreased carotenoid production, while it remained unaffected when exposed to AgNO_3_. These results may reflect differences in the fundamental biosynthetic pathways and regulation of these pigments. While chl *a* and chl *b* share several common steps, the final conversion to chl *b* depends on chlorophyll *a* oxygenase (CAO), whose activity can be differentially regulated under stress conditions to improve light harvesting. Carotenoids, on the other hand, are synthesized via a separate mevalonate pathway and are highly sensitive to oxidative stress [43]. Moreover, these differences in algal response to photosynthetic pigment production between AgNPs and AgNO_3_ treatments may be attributed to the differential effect of the nanoparticulate form of silver compared to Ag^+^ ions from AgNO_3_ treatment. The increase in chlorophylls observed after AgNO_3_ exposure may represent an early adaptive response aimed at compensating for Ag^+^-induced inhibition of photosynthetic electron transport downstream of PSII (*ET*_0_/*RC*). Previous studies have shown that AgNO_3_ disrupts the H^+^ pump thereby reducing energy production in algae [44]. This adaptive increase in chlorophyll content may be maintained because, in our previous study [11], AgNO_3_ strongly induced antioxidant enzymes, which likely mitigated oxidative damage and prevented extensive chlorophyll degradation. On the other hand, since carotenoids participate in energy dissipation in PSII, the significant decrease in their content only after the AgNP treatments suggests that nanoparticles reduce the ability of PSII to protect against photooxidation, which aligns with previous studies on the effect of AgNPs on duckweed *Spirodela polyrhiza* [45]. In addition, we have already shown that AgNP treatments cause a significant increase in ROS production [11], which may contribute to the degradation of carotenoids. Interestingly, AgNO_3_ exposure did not reduce carotenoid content, which could be related to the strongest activation of antioxidant enzymes observed with this treatment, leading to lower ROS accumulation. Alternatively, this could also suggest that the toxicity mechanism of AgNPs is more closely related to their surface reactivity and ROS generation, whereas the toxicity of AgNO_3_ may stem from the direct interactions of Ag^+^ ions with biomolecules.

Despite the different effects of AgNPs and AgNO_3_ on chl *a* and *b* content, all treatments caused a significant decrease in the photosynthetic rate, as well as in the maximum quantum yield of PSII (*F_v_*/*F_m_*), which is consistent with the decreased *F_v_*/*F_m_* values obtained when the alga *Poterioochromonas malhamensis* was exposed to AgNP-citrate and AgNO_3_ [46]. Although *F_v_*/*F_m_* was reduced, the *PI_abs_*, involving multiple levels of the photosynthetic process, showed a different response between treatments; namely, while AgNO_3_ treatment significantly decreased *PI_abs_*, exposure to AgNPs resulted in a moderate (AgNP-citrate) and significant (AgNP-CTAB) increase, which may indicate the activation of compensatory mechanisms and a more efficient reorganization of the photosynthetic apparatus [47,48]. Nevertheless, the observed decrease in photosynthetic rate shows that all silver treatments had a significant negative effect on the photosynthetic apparatus of *C. vulgaris*, while the decrease in *F_v_*/*F_m_* indicates photoinhibition, i.e., inhibition of electron transfer in photochemical reactions and damage to PSII reaction centers [49,50,51]. Under conditions of severe oxidative stress, degradation of PSII reaction centers and reduced electron transfer to plastoquinone may occur [52], which is consistent with the findings that treatments with AgNPs and AgNO_3_ induce strong oxidative stress in *C. vulgaris* [11]. Analysis of the energy flux through PSII showed that treatment with AgNO_3_ had a much stronger effect on these parameters than exposure to AgNPs. Although the absorption energy flux per reaction center was not significantly altered in any of the treatments, indicating a preserved ability of the antennae to absorb light, the trapping of excited energy in PSII was significantly decreased after exposure to AgNP-CTAB and AgNO_3_. In addition, AgNO_3_ treatment decreased *ET*_0_/*RC*, i.e., electron transport downstream of PSII), indicating inhibition of photochemical electron transfer and damage to the redox components of the photosystem. These findings are consistent with the results of other studies showing that Ag^+^ ions impair electron transport in thylakoid membranes [53,54]. On the other hand, the amount of energy dissipated as heat and fluorescence increased after all treatments, albeit only significantly when exposed to AgNO_3_, suggesting that Ag^+^ ions, which can also be released from AgNPs, stimulate the cells’ attempt to protect the photosynthetic apparatus from excess unused light energy through passive dissipation mechanisms [55,56,57]. These results are also confirmed by the *NPQ* values, which increased significantly after treatment with AgNP-CTAB and AgNO_3_. *NPQ* reflects the activation of photoprotective mechanisms, primarily the xanthophyll cycle and the Δ*pH* mechanism, which enable the dissipation of excess excitation energy and the prevention of photooxidative damage [55,57]. The significantly higher NPQ observed after AgNP-CTAB treatment can be attributed to the substantially greater nanoparticle dose required to reach EC_25_. Specifically, AgNP-CTAB was applied at a concentration of 0.895 mg L^−1^, which is nearly five times higher than that of AgNP-citrate (0.188 mg L^−1^), resulting in increased nanoparticle uptake [11]. This enhanced uptake is further promoted by the positive surface charge provided by the CTAB coating, which facilitates a stronger interaction with the negatively charged algal cell membranes. Consequently, more nanoparticles interact with photosynthetic complexes and intensify photo stress, thereby triggering a stronger NPQ response compared to citrate-coated AgNPs [11,58]. Additionally, all treatments led to an increase in the photochemical coefficient *Qp*, which indicates the proportion of open PSII centers. The largest increase was observed in the AgNP-CTAB treatment, while the AgNP-citrate and AgNO_3_ treatments caused an equal but less pronounced increase. The maintenance of open PSII centers despite reduced *F_v_*/*F_m_* values can be explained by a blockage of electron transfer downstream of PSII, resulting in unutilized excitation energy, which the cells compensate for by opening additional centers and increasing dissipation [59]. These results are consistent with other studies showing that the *QY* parameter can remain relatively stable during the early stages of stress, while parameters such as *NPQ* and *Qp* are more sensitive to changes in photosynthetic dynamics [48,60]. Overall, the chl *a* fluorescence results indicate the occurrence of photoinhibition and electron transport disturbances, especially after exposure to AgNO_3,_ but also very clearly to AgNP-CTAB, accompanied by concomitant activation of protective mechanisms such as NPQ. These changes are most likely a consequence of the pronounced oxidative stress induced by Ag^+^ ions, which are either added in the form of AgNO_3_ salt or dissociated from AgNPs. Released Ag^+^ ions can replace copper in plastocyanin, bind to sulfhydryl groups, and disrupt the structure and function of thylakoid proteins [61], and consequently lead to damage of PSII reaction centers, impaired chlorophyll synthesis, and reduced electron transport efficiency, resulting in an overall significant decrease in photosynthetic activity and increased oxidative burden.

To better understand the molecular basis of the observed physiological effects, we complemented the analysis of photosynthetic parameters with the profiling of the differential expression of photosynthesis-related genes and proteins. This integrative approach, applied to AgNPs with different surface coatings as well as to AgNO_3_ at comparable phytotoxic concentrations (EC_25_), provides a more comprehensive insight into how AgNPs’ surface chemistry modulates molecular responses in *C. vulgaris*. Such combined molecular and physiological analyses are still relatively rare and thus provide a better understanding of AgNP-induced phytotoxicity mechanisms. AgNPs can have a significant impact on protein abundance and enzyme activity through direct interaction or indirectly through induction of ROS production, which can oxidize proteins and damage DNA molecules, thereby reducing transcription [8]. In our study, exposure to AgNP-citrate and AgNO_3_ resulted in a similar number of proteins with altered expression (21 and 20, respectively), most of which showed increased expression, while treatment with AgNP-CTAB induced changes in the expression of 27 proteins, which were mainly downregulated. These differences in the regulation of protein expression can be directly linked to the effects of the different AgNPs coatings. Indeed, the results of the study conducted on *C. vulgaris* [22] indicate that negatively charged AgNPs (such as AgNP-citrate) primarily affect mitochondrial proteins, thereby destabilizing important metabolic pathways such as oxidative phosphorylation. Cells could compensate for this by increasing the synthesis of photosynthesis-related proteins to enhance energy production, as confirmed in our study. In contrast, positively charged AgNPs, such as AgNP-CTAB, have been shown to primarily target ribosome-related proteins and can interfere with protein synthesis and transcription pathways [22], with a direct consequence in the form of significant downregulation of proteins, which we found.

Exposure to both types of AgNPs resulted in a majority of differentially expressed proteins related to photosynthesis, albeit with opposite effects; namely, AgNP-citrate induced the expression of the majority of proteins, while AgNP-CTAB decreased the expression of all proteins. This can be correlated with the results of the maximum quantum yield of PSII, which was significantly higher with AgNP-citrate compared to AgNP-CTAB. Moreover, a decrease in the expression of the large subunit of RuBisCo was observed only after treatment with AgNP-CTAB, while AgNP-citrate upregulated the expression of the small subunit, which, together with the results of photosynthetic parameters, indicates more severe damage to the photosynthetic apparatus after treatment with AgNP-CTAB compared to AgNP-citrate. In addition, AgNP-citrate increased the expression of proteins involved in reactions in PSI and PSII, such as the binding of chl *a* and *b* (chlorophyll *a*-*b* binding protein), oxygen evolution or water photolysis (PsbO2 protein), and the formation of complexes with ferredoxin and ferredoxin oxidoreductase in the electron transport chain, which increases NADPH production for further carbon fixation (PSI reaction center subunit 4 protein), contributing to the maintenance of functional photosynthesis. Similar results were reported when the alga *C. reinhardtii* was exposed to PEG-coated AgNPs, where most of the upregulated proteins were involved in photosynthesis and the Calvin-Benson cycle [62]. On the other hand, all the above proteins were downregulated after treatment with AgNP-CTAB, indicating inhibition of water photolysis and consequently oxygen release and ferredoxin activity. The observed differences in protein expression profiles between AgNP-CTAB and AgNP-citrate treatments may be attributed to the higher dose, as well as the enhanced cellular uptake facilitated by the positive surface charge of CTAB-coated AgNPs, which likely intensified interactions with photosynthetic structures and contributed to the observed inhibitory effects. Furthermore, although AgNO_3_ resulted in the smallest number of differentially expressed proteins within the Photosynthesis group, among which only two chlorophyll *a*-*b* binding proteins showed decreased expression, it caused the most pronounced reduction in photosynthetic parameters, including *F_v_*/*F_m_*, *PI_abs_* and electron transport efficiency. This discrepancy likely stems from the direct effects of Ag^+^ ions on PSII functionality, which can occur rapidly through interaction with key components of the thylakoid membrane, such as the plastocyanin and sulfhydryl groups of PSII proteins, impairing photosynthetic performance without significant changes in protein abundance [53].

Disruption of photosynthesis consequently impairs cellular energy production [45]. To compensate for lowered energy levels induced by disrupted photosynthesis after AgNP-citrate treatment, the algal cells increased the expression of proteins from the Carbohydrate Metabolism group, such as malate dehydrogenase, phosphoglycerate kinase, and NADP-dependent oxidoreductase. Malate dehydrogenase, an enzyme involved in the citric acid cycle [63], plays an important role under stress conditions, including oxidative stress, when algal cells increase lipid synthesis for the storage of energy [64], which could be used to activate antioxidant defenses [65]. The increased expression of phosphoglycerate kinase, which is involved in glycolysis, and NADP-dependent oxidoreductases also suggests an increased breakdown of molecules to increase energy production, likely as a compensatory response to reduced energy production by photosynthesis. The same proteins were upregulated upon exposure to AgNO_3_. On the other hand, AgNP-CTAB treatment resulted in downregulation of proteins involved in glycolysis (fructose-bisphosphate aldolase, glyceraldehyde-3-phosphate dehydrogenase, and phosphoglycerate kinase) and the citric acid cycle (malate dehydrogenase), suggesting that the algae slowed down their metabolism as a survival strategy under stress conditions [66]. Indeed, proteomic analysis of the alga *Pyropia haitanensis* under elevated salinity conditions showed a significant reduction in the expression of many proteins related to glycolysis and the pentose phosphate pathway compared to the controls [67]. Furthermore, a study on the alga *Microcystis aeruginosa* also demonstrated that exposure to AgNPs leads to reduced expression of proteins related to carbohydrate metabolism [68].

In addition to proteins directly related to photosynthesis, differentially expressed proteins involved in defense and stress response, signal transduction, transcription, and translation, as well as cellular structure, were also identified. Their altered expression indicates a systemic cellular response to stress, which is consistent with our previous research demonstrating the occurrence of oxidative stress in *C. vulgaris* after exposure to silver nanoparticles and ions [11].

Analysis of the expression of key chloroplast genes associated with photosynthesis revealed that treatment with AgNP-CTAB caused a slightly stronger downregulation of *atpE*, *psbA*, *psaA*, *psbB*, *petD*, and *psaB* compared to AgNP-citrate. This difference could be related to the slightly greater DNA damage observed after AgNP-CTAB exposure in our previous study [11], but could also result from its higher EC_25_ concentration and the consequently greater number of nanoparticles interacting with the photosynthetic machinery. Moreover, this expression pattern correlates with the results of differential protein expression, in which proteins related to photosynthesis (e.g., ATP synthase and photosystem complex proteins) decreased significantly and most strongly after treatment with AgNP-CTAB. The effects of AgNO_3_ on gene expression were intermediate when compared to both types of AgNPs, which suggests that Ag^+^ ions significantly contribute to DNA damage and photosynthetic disruption. Furthermore, all treatments led to decreased expression of the *psbA* and *atpE* genes, both of which play essential roles in photosynthetic performance. The *psbA* gene and its protein product, the D1 protein, are well-known sensitive indicators of oxidative damage to PSII [69], and its decreased expression can lead to impaired photochemical activity and degradation of the photosynthetic apparatus. Similarly, decreased expression of the *atpE* gene, which encodes the F_0_ component of ATP synthase, could lead to reduced energy availability for photochemical processes, possibly explaining the observed reduction in photosynthetic efficiency parameters such as *F_v_*/*F_m_*. On the other hand, the stable expression of the *atpF* gene across all treatments suggests that some ATP synthase subunits are less sensitive, which may be due to the adaptive or compensatory mechanisms of algae under stress conditions [70].

These findings are consistent with previous reports showing that AgNPs induce oxidative stress and damage photosynthetic complexes in algae and plants [12,71]. In the case of AgNP-CTAB, such effects may be further enhanced by the cationic nature of the CTAB coating, which facilitates interactions with chloroplast membranes, as well as the larger number of nanoparticles required to achieve EC_25_, further increasing their harmful potential [72].

## 5. Conclusions

This study shows that AgNPs exert coating-dependent effects on photosynthesis in *C. vulgaris* that are distinctly different from the effects of ionic silver. Both AgNP types altered pigment composition by increasing chl *b* content and reducing carotenoids, whereas AgNO_3_ increased both chlorophylls without affecting carotenoids. All treatments led to reduced photosynthetic efficiency, with AgNP-CTAB and AgNO_3_ exhibiting the most pronounced inhibition of PSII activity and electron transport. Proteome and transcriptome analyses showed that AgNP-CTAB triggered stronger downregulation of photosynthesis-related genes and proteins, while AgNP-citrate elicited comparatively milder molecular responses. These results emphasize the crucial role of surface chemistry, as the cationic CTAB coating enhances cellular uptake and increases toxicity. Overall, both nanoparticulate and ionic forms of silver interfere with algal photosynthesis primarily through oxidative stress and PSII inhibition, with coating-dependent variations in nanoparticle stability, internalization, and bioavailability determining the severity of their effects. However, the magnitude and nature of these effects are strongly influenced by nanoparticle surface properties, highlighting the necessity of considering surface functionalization when assessing the ecological risks of engineered nanomaterials in aquatic environments.

## Figures and Tables

**Table 1:** Primer sequences and amplification efficiencies for target genes analyzed using quantitative PCR. Efficiencies were calculated using Mic qPCR Analysis Software 2.12.7 Bio Molecular Systems, Upper Coomera QLD, Australia).

Gene	Locus Tag	5’ → 3’ Sequence (Forward/Reverse)	Primer Efficiency
*atpE*	ChvulCp147	ACCGCCCATAAGTGCTACAG/TCCCACTAATACGGGTCAAATGG	0.94
*atpF*	ChvulCp022	GTTGCCCGTTGATCTGCTTC/AGCCGTGGTATTGGCTATCG	0.97
*petD*	ChvulCp077	CACCAATTGCTGCTGGGTCT/TTATGGTGAACCAGCGTGGC	0.72
*psaA*	ChvulCp091	GGGAACAGTAAGTGCAAACGG/ACTTGTGAGGATTGTGCCCAT	0.95
*psaB*	ChvulCp093	AAGGTGCTCTTGACGCTCG/TCACATGTACCGCCACGAC	0.95
*psbA*	ChvulCp007	CAGCTGGAGCTTCAACAACC/GGCTGACATTATCAACCGTGC	0.98
*psbB*	ChvulCp090	GGAACATCCGCACGAACAAC/AGTTGGCTGGTTAGGTCACG	0.97
*CYP*	/	CTTCCGCGCTCTGTGCACTG/GCCGTAGATGGACTTGCCGCC	0.97

Gene description: *atpE*—ATP synthase CF1 epsilon subunit; *atpF*—ATP synthase CF0 B chain; *petD*—cytochrome *b6f* complex subunit IV; *psaA*—photosystem I P700 chlorophyll *a* apoprotein A1; *psaB*—photosystem I P700 chlorophyll *a* apoprotein A2; *psbA*—photosystem II protein D1; *psbB*—photosystem II 47 kDa protein; *CYP*—cyclophilin.

**Table 2:** Differentially expressed proteins in *C. vulgaris* cells exposed to AgNP-citrate (0.188 mg L^−1^), AgNP-CTAB (0.895 mg L^−1^), and AgNO_3_ (0.130 mg L^−1^) for 72 h. Upward arrow (↑) indicates increased, downward arrow (↓) decreased, and equal sign (=) unchanged expression compared to the control. pI—isoelectric point, M—molecular mass.

Spot Label	Protein Name	pI	M (kDa)	Differential Expression		
				AgNP-Citrate	AgNP-CTAB	AgNO_3_
**Photosynthesis**						
1	Beta carbon anhydrase	5.74	51.9	↓	↓	=
3	Protein PsbP	9.22	26.4	↑	=	=
5	Ribulose-1,5-bisphosphate carboxylase, large subunit	5.99	52.5	=	↓	=
11 28	Ribulose-1,5-bisphosphate carboxylase, small subunit	9.32 9.44	20.12 20.04	↑ =	= =	↑ ↑
12	Reaction center protein, subunit 2	9.71	20.72	↑	=	=
15 25 34 6 26	Chlorophyll *a*-*b* binding protein	6.77 4.85 6.19 5.93 5.93	31.36 26.85 30.70 22.94 22.94	= ↓ = ↑ ↑	↓ ↓ ↓ ↓ ↓	↓ = ↓ = =
27	Protein PsbO2	5.16	30.72	↑	↓	=
29	Photosystem I reaction center protein, subunit 4	9.98	10.89	↑	=	=
**Electron Transport and Energy Production**						
10	ATP synthase gamma chain	8.95	39.38	=	↓	=
22	ATP synthase, beta subunit	4.93	51.64	↓	↑	↑
**Carbohydrate Metabolism**						
8	Fructose bisphosphate aldolase	6.49	40.95	=	↓	=
13 16	Glyceraldehyde-3-phosphate dehydrogenase	9.05 5.91	43.20 36.07	=	↓ ↓	↓ ↑
19	Malate dehydrogenase	5.74	34.77	↑	↓	↑
20	Phosphoglycerate kinase	6.93	48.48	↑	↓	↑
36	NADP- dependent oxidoreductase	7.57	33.31	↑	=	↑
**Defense and Stress Response**						
21	Heat shock protein 70B	5.15	72.08	=	↓	=
31	Antifreeze	9.68	27.43	↑	↑	↑
32	Superoxide dismutase	8.63	26.06	=	=	↑
35	Lactate dehydrogenase	4.98	24.34	↑	=	↑
**Signal Transduction**						
4 9 17 18	Calcium-dependent protein kinase and calmodulin	8.78 7.71 7.71 7.71	40.69 38.49 38.49 38.49	↓ ↓ = =	↓ ↓ ↓ ↓	= ↓ = =
23	Rhodanase	4.63	39.45	↓	=	↑
30	Voltage-dependent ion channel	8.51	28.73	↑	↓	=
**Transcription and Translation Processes**						
7	Translational GTPase	8.73	50.82	=	↓	↓
37 2	Elongation factor Tu	5.36 5.36	44.9 44.9	=	↓ ↓	=
33	RRM domain-containing protein	5.07	25.57	↑	=	↑
**Storage protein**						
14	Cupin type 1	9.26	21.9	↑	=	↑
**Mechanical Support of the Cell**						
24	Actin	5.3	41.77	=	↑	↑

## Data Availability

The data that support the findings of this study are available from the corresponding author upon reasonable request.

## Supplementary Materials

The following supporting information can be downloaded at https://www.mdpi.com/article/10.3390/toxics13080627/s1: Figure S1: UV/vis absorption spectra show absorbance peaks marked by arrows (A and E), while AgNP-citrate (B–D) and AgNP-CTAB (F–H) particles are visualized using transmission electron microscopy (TEM). Panels B and F display particles in bright field, C and G present silver element distribution maps, and D and H show EDX spectra, with arrows indicating the characteristic X-ray peaks of silver. Four independent replicates were analyzed for each stock suspension (n = 4). Scale bar represents 500 nm; Table S1: Parameters of peptide analysis using Agilent 1290 Infinity II ultra-high-performance liquid chromatography (UHPLC).; Table S2: Parameters of peptide analysis using Agilent 6545 Q-TOF mass spectrometer (MS).; Table S3: The physicochemical properties of AgNP-citrate and AgNP-CTAB in their stock suspensions include the hydrodynamic diameter (*d*_H_) derived from volume-based size distribution, ζ potential, total silver concentration, and the percentage of silver present as ions (Ag^+^).; Table S4: Differentially expressed proteins, their cellular localization and biological and molecular function in *C. vulgaris* cells exposed to AgNP-citrate (0.188 mg L^−1^), AgNP-CTAB (0.895 mg L^−1^), and AgNO_3_ (0.130 mg L^−1^) for 72 h. Upward arrow (↑) indicates increased, downward arrow (↓) decreased, and equal sign (=) unchanged expression compared to the control. pI—isoelectric point, M—molecular mass.

## References

[1] Temizel-Sekeryan S., Hicks A.L. (2020). Global environmental impacts of silver nanoparticle production methods supported by life cycle assessment. Resour. Conserv. Recycl..

[2] Pulit-Prociak J., Banach M. (2016). Silver nanoparticles—A material of the future…?. Open Chem..

[3] Gherasim O., Puiu R.A., Bîrcă A.C., Burdușel A.-C., Grumezescu A.M. (2020). An updated review on silver nanoparticles in biomedicine. Nanomaterials.

[4] Dawadi S., Katuwal S., Gupta A., Lamichhane U., Thapa R., Jaisi S., Lamichhane G., Bhattarai D.P., Parajuli N. (2021). Current research on silver nanoparticles: Synthesis, characterization, and applications. J. Nanomater..

[5] Rana A., Parmar A.S. (2023). Re-exploring silver nanoparticles and its potential applications. Nanotechnol. Environ. Eng..

[6] Wu L., Zhang J., Watanabe W. (2011). Physical and chemical stability of drug nanoparticles. Adv. Drug Deliv. Rev..

[7] Baig N., Kammakakam I., Falath W. (2021). Nanomaterials: A review of synthesis methods, properties, recent progress, and challenges. Mater. Adv..

[8] Biba R., Štefanić P.P., Cvjetko P., Tkalec M., Balen B., Abd-Elsalam K.A. (2021). Silver nanoparticles phytotoxicity mechanisms. Silver Nanomaterials for Agri-Food Applications.

[9] Rónavári A., Bélteky P., Boka E., Zakupszky D., Igaz N., Szerencsés B., Pfeiffer I., Kónya Z., Kiricsi M. (2021). Polyvinyl-Pyrrolidone-coated silver nanoparticles—The colloidal, chemical, and biological consequences of steric stabilization under biorelevant conditions. Int. J. Mol. Sci..

[10] Filipczak P., Borkowski M., Chudobinski P., Bres S., Matusiak M., Nowaczyk G., Kozanecki M. (2020). Sodium citrate stabilized AgNPs under thermal treatment, electron-beam and laser irradiations. Radiat. Phys. Chem..

[11] Komazec B., Cvjetko P., Balen B., Letofsky-Papst I., Lyons D.M., Peharec Štefanić P. (2023). The occurrence of oxidative stress induced by silver nanoparticles in *Chlorella vulgaris* depends on the surface-stabilizing agent. Nanomaterials.

[12] Biba R., Košpić K., Komazec B., Markulin D., Cvjetko P., Pavoković D., Paharec Štefanić P., Tkalec M., Balen B. (2021). Surface coating-modulated phytotoxic responses of silver nanoparticles in plants and freshwater green algae. Nanomaterials.

[13] Fabrega J., Luoma S.N., Tyler C.R., Galloway T.S., Lead J.R. (2011). Silver nanoparticles: Behaviour and effects in the aquatic environment. Environ. Int..

[14] Shalaby E. (2011). Algae as promising organisms for environment and health. Plant Signal. Behav..

[15] Turan N.B., Erkan H.S., Engin G.O., Bilgili M.S. (2019). Nanoparticles in the aquatic environment: Usage, properties, transformation and toxicity—A review. Process Saf. Environ. Protect..

[16] Field C.B., Behrenfeld M.J., Randerson J.T., Falkowski P. (1998). Primary production of the biosphere: Integrating terrestrial and oceanic components. Science.

[17] Aljaghoub H., Alasad S., Alashkar A., AlMallahi M., Hasan R., Obaideen K., Alami A.H. (2023). Comparative analysis of various oxygen production techniques using multi-criteria decision-making methods. Int. J. Thermofluids.

[18] Xiu Z., Zhang Q., Puppala H.L., Colvin V.L., Alvarez P.J.J. (2012). Negligible particle-specific antibacterial activity of silver nanoparticles. Nano Lett..

[19] He D., Dorantes-Aranda J.J., Waite T.D. (2012). Silver nanoparticle—Algae interactions: Oxidative dissolution, reactive oxygen species generation and synergistic toxic effects. Environ. Sci. Technol..

[20] Hazeem L.J., Kuku G., Dewailly E., Slomianny C., Barras A., Hamdi A., Boukherroub R., Culha M., Bououdina M. (2019). Toxicity effect of silver nanoparticles on photosynthetic pigment content, growth, ROS production and ultrastructural changes of microalgae *Chlorella vulgaris*. Nanomaterials.

[21] Von Moos N., Slaveykova V.I. (2014). Oxidative stress induced by inorganic nanoparticles in bacteria and aquatic microalgae—State of the art and knowledge gaps. Nanotoxicology.

[22] Zhang J., Shen L., Xiang Q., Ling J., Zhou C., Hu J., Chen L. (2020). Proteomics reveals surface electrical property-dependent toxic mechanisms of silver nanoparticles in *Chlorella vulgaris*. Environ. Pollut..

[23] Oukarroum A., Bras S., Perreault F., Popovic R. (2012). Inhibitory effects of silver nanoparticles in two green algae, *Chlorella vulgaris* and *Dunaliella tertiolecta*. Ecotoxicol. Environ. Saf..

[24] Dewez D., Oukarroum A. (2012). Silver nanoparticles toxicity effect on photosystem II photochemistry of the green alga *Chlamydomonas reinhardtii* treated in light and dark conditions. Toxicol. Environ. Chem..

[25] Pham T.-L.L. (2019). Effect of silver nanoparticles on tropical freshwater and marine microalgae. J. Chem..

[26] Wang Z., Quik J.T.K., Song L., Van Den Brandhof E.J., Wouterse M., Peijnenburg W.J.G.M. (2015). Humic substances alleviate the aquatic toxicity of polyvinylpyrrolidone-coated silver nanoparticles to organisms of different trophic levels. Environ. Toxicol. Chem..

[27] Bischoff H.W., Bold H.C. (1963). Some Soil Algae from Enchanted Rock and Related Algal Species.

[28] OECD (2011). Test no. 201: Freshwater alga and cyanobacteria, growth inhibition test. OECD Guidelines for the Testing of Chemicals, Section 2.

[29] Jarantow S.W., Pisors E.D., Chiu M.L. (2023). Introduction to the use of linear and nonlinear regression analysis in quantitative biological assays. Curr. Protoc..

[30] Schumann R., Häubner N., Klausch S., Karsten U. (2005). Chlorophyll extraction methods for the quantification of green microalgae colonizing building facades. Int. Biodeterior. Biodegrad..

[31] Jeffrey S.W., Humphrey G.F. (1975). New spectrophotometric equations for determining chlorophylls *a*, *b*, *c1* and *c2* in higher plants, algae and natural phytoplankton. Biochem. Physiol. Pflanzen.

[32] Dere Ş., Güneş T., Sivaci R. (1998). Spectrophotometric determination of chlorophyll—*a*, *b* and total carotenoid contents of some algae species using different solvents. Turk. J. Bot..

[33] Pavoković D., Križnik B., Krsnik-Rasol M. (2012). Evaluation of protein extraction methods for proteomic analysis of non-model recalcitrant plant tissues. Croat. Chem. Acta.

[34] Peharec Štefanić P., Cvjetko P., Biba R., Domijan A.-M.M., Letofsky-Papst I., Tkalec M., Šikić S., Cindrić M., Balen B. (2018). Physiological, ultrastructural and proteomic responses of tobacco seedlings exposed to silver nanoparticles and silver nitrate. Chemosphere.

[35] Tega J., Soh N., Awatif C., Azman S.N.A.N., San C.T., Jusoh M. (2020). Comparison of seven housekeeping genes expression in microalgae (*Chlorella vulgaris*) grown under nitrogen limited condition. Malays. Appl. Biol..

[36] Pfaffl M.W. (2004). Quantification strategies in real-time PCR. A–Z of Quantitative PCR.

[37] Vandesompele J., De Preter K., Pattyn F., Poppe B., Van Roy N., De Paepe A., Speleman F. (2002). Accurate normalization of real-time quantitative rt-PCR data by geometric averaging of multiple internal control genes. Genome Biol..

[38] Ma W., Liu L.-N., Wang Q., Duanmu D., Qiu B.-S. (2023). Editorial: Algal photosynthesis. Front. Microbiol..

[39] Chen Z., Wu W., Wen Y., Zhang L., Wu Y., Farid M.S., El-Seedi H.R., Capanoglu E., Zhao C. (2023). Recent advances of natural pigments from algae. Food Prod. Process. Nutr..

[40] Larkum A.W. (2016). Photosynthesis and light harvesting in algae. The Physiology of Microalgae.

[41] Wang Z., Guan Y., Xu Y., Ding J., Ma J., Ma H., Terry N. (2019). Effects of nanoparticles on algae: Adsorption, distribution, ecotoxicity and fate. Appl. Sci..

[42] Kaur M., Saini K.C., Ojah H., Sahoo R., Gupta K., Kumar A., Bast F. (2022). Abiotic stress in algae: Response, signaling and transgenic approaches. J. Appl. Phycol..

[43] Xu P., Yu J., Ma R., Ji Y., Hu Q., Mao Y., Ding C., Li Z., Ge S., Deng W.-W. (2024). Chlorophyll and carotenoid metabolism varies with growth temperatures among tea genotypes with different leaf colors in *Camellia sinensis*. Int. J. Mol. Sci..

[44] Loseva N.L., Alyabyev A.J., Gordon L.K., Andreyeva I.N., Kolesnikov O.P., Ponomareva A.A., Kemp R.B. (2007). The effect of AgNO_3_ on the bioenergetic processes and the ultrastructure of *Chlorella* and *Dunaliella* cells exposed to different saline conditions. Thermochim. Acta.

[45] Jiang H.S., Yin L.Y., Ren N.N., Zhao S.T., Li Z., Zhi Y., Shao H., Li W., Gontero B. (2017). Silver nanoparticles induced reactive oxygen species via photosynthetic energy transport imbalance in an aquatic plant. Nanotoxicology.

[46] Li M., Liu W., Slaveykova V.I. (2020). Effects of mixtures of engineered nanoparticles and metallic pollutants on aquatic organisms. Environments.

[47] Živčák M., Brestič M., Olšovská K., Slamka P. (2008). Performance index as a sensitive indicator of water stress in *Triticum aestivum* L.. Plant Soil. Environ..

[48] Oukarroum A., Schansker G., Strasser R.J. (2009). Drought stress effects on photosystem I content and photosystem II thermotolerance analyzed using chl *a* fluorescence kinetics in barley varieties differing in their drought tolerance. Physiol. Plant.

[49] Zushi K., Kajiwara S., Matsuzoe N. (2012). Chlorophyll *a* fluorescence OJIP transient as a tool to characterize and evaluate response to heat and chilling stress in tomato leaf and fruit. Sci. Hortic..

[50] Zhou G., Xu L., Wang H., Sun A., Wang Y., Li X., Jiang R. (2023). Different responses of *Chlorella vulgaris* to silver nanoparticles and silver ions under modulation of nitric oxide. Environ. Sci. Pollut. Res..

[51] Yan J., Zou Y., Zhang F., Zhang S., Huang X., Benoit G. (2022). Growth, ROS accumulation site, and photosynthesis inhibition mechanism of *Chlorella vulgaris* by triclosan. Environ. Sci. Pollut. Res..

[52] Almeida A.C., Gomes T., Langford K., Thomas K.V., Tollefsen K.E. (2019). Oxidative stress potential of the herbicides bifenox and metribuzin in the microalgae *Chlamydomonas reinhardtii*. Aquat. Toxicol..

[53] Cvjetko P., Balen B., Pavoković D., Šikić S., Peharec Štefanić P., Tkalec M., Biba R. (2021). Silver nanoparticles affect germination and photosynthesis in tobacco seedlings. Acta Bot. Croat..

[54] Pociecha E., Gorczyca A., Dziurka M., Matras E., Oćwieja M. (2021). Silver Nanoparticles and silver ions differentially affect the phytohormone balance and yield in wheat. Agriculture.

[55] Koletti A., Skliros D., Dervisi I., Roussis A., Flemetakis E. (2025). Oxidative stress responses in microalgae: Modern insights into an old topic. Appl. Microbiol..

[56] Peharec Štefanić P., Košpić K., Lyons D.M., Jurković L., Balen B., Tkalec M. (2021). Phytotoxicity of silver nanoparticles on tobacco plants: Evaluation of coating effects on photosynthetic performance and chloroplast ultrastructure. Nanomaterials.

[57] Demmig-Adams B., Adams W.W., Barker D.H., Logan B.A., Bowling D.R., Verhoeven A.S. (1996). Using chlorophyll fluorescence to assess the fraction of absorbed light allocated to thermal dissipation of excess excitation. Physiol. Plant.

[58] Huang D., Dang F., Huang Y., Chen N., Zhou D. (2022). Uptake, translocation, and transformation of silver nanoparticles in plants. Environ. Sci. Nano.

[59] Oukarroum A., Polchtchikov S., Perreault F., Popovic R. (2012). Temperature Influence on silver nanoparticles inhibitory effect on photosystem II photochemistry in two green algae, *Chlorella vulgaris* and *Dunaliella tertiolecta*. Environ. Sci. Pollut. Res..

[60] Zuo G. (2025). Non-photochemical quenching (NPQ) in photoprotection: Insights into NPQ levels required to avoid photoinactivation and photoinhibition. New Phytol..

[61] Kircheva N., Angelova S., Dobrev S., Petkova V., Nikolova V., Dudev T. (2023). Cu^+^/Ag^+^ competition in type I copper proteins (T1Cu). Biomolecules.

[62] Lindgren A.L. (2014). The Effects of Silver Nitrate and Silver Nanoparticles on *Chlamydomonas reinhardtii*: A Proteomic Approach. Master’s Thesis.

[63] Cavalcanti J.H.F., Esteves-Ferreira A.A., Quinhones C.G.S., Pereira-Lima I.A., Nunes-Nesi A., Fernie A.R., Araújo W.L. (2014). Evolution and functional implications of the tricarboxylic acid cycle as revealed by phylogenetic analysis. Genome Biol. Evol..

[64] Shi T.-Q., Wang L.-R., Zhang Z.-X., Sun X.-M., Huang H. (2020). Stresses as first-line tools for enhancing lipid and carotenoid production in microalgae. Front. Bioeng. Biotechnol..

[65] A. Lal M. (2018). Respiration. Plant Physiology, Development and Metabolism.

[66] Roychoudhury A. (2022). Photosynthesis and Respiratory Cycles During Environmental Stress Response in Plants.

[67] Wen J., Wang W., Xu K., Ji D., Xu Y., Chen C., Xie C. (2020). Comparative analysis of proteins involved in energy metabolism and protein processing in *Pyropia haitanensis* at different salinity levels. Front. Mar. Sci..

[68] Qian H., Zhu K., Lu H., Lavoie M., Chen S., Zhou Z., Deng Z., Chen J., Fu Z. (2016). Contrasting silver nanoparticle toxicity and detoxification strategies in *Microcystis aeruginosa* and *Chlorella vulgaris*: New insights from proteomic and physiological analyses. Sci. Total Environ..

[69] Mulo P., Sicora C., Aro E.-M. (2009). Cyanobacterial PsbA gene family: Optimization of oxygenic photosynthesis. Cell. Mol. Life Sci..

[70] Kong I.C., Ko K.S., Koh D.C. (2020). Evaluation of the effects of particle sizes of silver nanoparticles on various biological systems. Int. J. Mol. Sci..

[71] Liu W., Majumdar S., Li W., Keller A.A., Slaveykova V.I. (2020). Metabolomics for early detection of stress in freshwater alga *Poterioochromonas malhamensis* exposed to silver nanoparticles. Sci. Rep..

[72] Oćwieja M., Barbasz A., Wasilewska M., Smoleń P., Duraczyńska D., Napruszewska B.D., Kozak M., Węgrzynowicz A. (2024). Surface charge-modulated toxicity of cysteine-stabilized silver nanoparticles. Molecules.

