# Supplementary material for: Multi-Parameter Analysis of Photosynthetic and Molecular Responses in Chlorella vulgaris Exposed to Silver Nanoparticles and Ions
Source: Toxics. 2025 Jul 26;13(8):627. doi: 10.3390/toxics13080627 (PMC12389777; doi:10.3390/toxics13080627)
Supplement: Supplementary file 1 [file toxics-13-00627-s001.zip › Supplemental Tables and Figure_proofread.pdf]

Table S1. Parameters of peptide analysis by Agilent 1290 Infinity II ultra-high-performance liquid chromatography (UHPLC).

Analytical column	*Agilent AdvanceBio Peptide Map,* 1.0 × 150 mm	
Column temperature	60 °C	
Autosampler temperature	10 °C	
Flow	0.1 mL min $^{-1}$	
Injection volume	8 μL	
Mobile phase A	0.1% FA / H_2O	
Mobile phase B	0.1% FA / 95% ACN	
Gradient	Time (min)	%B
	0	3
	10	40
	14	90
	16	90
	18	3
	21	3

Table S2. Parameters of peptide analysis by Agilent 6545 Q-TOF mass spectrometer (MS).

Ionization method	ESI with positive ionization
Gas temperature (N_2)	325 °C
Gas flow	13 L min $^{-1}$
Nebulizer	35 psi
Sheath gas temperature	275 °C
Sheath gas flow	12 L min $^{-1}$
Capillary voltage	4000 V
Nozzle voltage	0V
Fragmentor voltage	175 V
Skimmer voltage	65 V
Octopole RF Vpp	750 V
MS recording range (m/z)	100 - 2000
MS acquisition speed (spectra/s)	3
Auto MS/MS recording range (m/z)	100 - 2000
MS/MS acquisition speed (spectra/s)	2
Insulation width	Medium (~4 amu)
Maximum number of precursors per cycle	10
Precursor sorting	First by charge, then by intensity
Collision energy	3.1 × (*m*/*z*)/100+1 for charge 2^+<br>3.6 × (*m*/*z*)/100-4.8 for charge 3^+
MS/MS threshold (absolute number)	3000
Threshold for MS/MS (rel.) (%)	0.001
Dynamic shutdown	Recording 2 spectra, and switching off for a period of 0.05 min
MS/MS accumulation time frame	25,000 " *counts* " per spectrum
Reference ions during analysis	*m*/*z* 121.050873 (purines)<br>*m*/*z* 922.009798 (HP-921)

Table S3. The physicochemical properties of AgNP-citrate and AgNP-CTAB in their stock suspensions include the hydrodynamic diameter (d_H) derived from volume-based size distribution, ζ potential, total silver concentration, and the percentage of silver present as ions (Ag^+).

**Property**	**AgNP-citrate**	**AgNP-CTAB**
Hydrodynamic diameter (d_H), nm	43.1 ± 0.8	80.1 ± 1.5
ζ potential, mV	-45.62 ± 2.68	39.89 ± 1.79
Concentration, mg L^{-1}	99.8	89.2
Ag^+, %	0.5	0.5

Table S4. Differentially expressed proteins, their cellular localization and biological and molecular function in *C. vulgaris* cells exposed to AgNP-citrate (0.188 mg L^{-1}), AgNP-CTAB (0.895 mg L^{-1}), and $AgNO_3$ (0.130 mg L^{-1}) for 72 hours. Upward arrow (↑) indicates increased, downward arrow (↓) decreased, and equal sign (=) unchanged expression compared to the control. pI – isoelectric point, M – molecular mass.

Spot label	Protein name	pI	M (kDa)	Subcellular localization	Biological process	Molecular function	Differential expression		
							AgNP-citrate	AgNP-CTAB	$AgNO_3$
Photosynthesis									
1	Beta carbon anhydrase	5.74	51.9	Chloroplast stroma	Photosynthesis	Increasing CO2 concentration around the enzyme RuBisCO, carbonic anhydrase / metalloenzyme	↓	↓	=
3	Protein PsbP	9.22	26.4	Thylakoid membrane (PSII)	Photosynthesis, PSII	Obtaining oxygen	↑	=	=
5	Ribulose-1,5-bisphosphate carboxylase, large subunit	5.99	52.5	Thylakoid membrane of chloroplast	Photosynthesis, photorespiration	CO_2 fixation	=	↓	=
11<br>28	Ribulose-1,5-bisphosphate carboxylase, small subunit	9.32<br>9.44	20.12<br>20.04	Thylakoid membrane of chloroplast	Photosynthesis, photorespiration	CO_2 fixation	↑<br>=	=<br>=	↑<br>↑
12	Reaction center protein, subunit 2	9.71	20.72	Thylakoid membrane of chloroplast	Photosynthesis	Formation of complex with ferredoxin and ferredoxin–NADP+ reductase	↑	=	=
15<br>25<br>34<br>6<br>26	Chlorophyll *a-b* binding protein	6.77<br>4.85<br>6.19<br>5.93<br>5.93	31.36<br>26.85<br>30.70<br>22.94<br>22.94	Thylakoid membrane of chloroplast (PSI and PSII)	Photosynthesis	Chlorophyll binding	=<br>↓<br>=<br>↑<br>↑	↓<br>↓<br>↓<br>↓<br>↓	↓<br>=<br>↓<br>=<br>=

Table S4. (continued)

27	Protein PsbO2	5.16	30.72	Thylakoid membrane of chloroplast	Photosynthesis	Oxygen evolution	↑	↓	=
29	Photosystem I reaction center protein, subunit 4	9.98	10.89	Thylakoid membrane of chloroplast (PSI)	Photosynthesis	Facilitates the connection of ferredoxin with PSI and ferredoxin-NADP oxidoreductase	↑	=	=
Electron Transport and Energy Production									
10	ATP synthase gamma chain	8.95	39.38	Thylakoid membrane of chloroplast	ATP synthesis, ion transport	Translocase	=	↓	=
22	ATP synthase, beta subunit	4.93	51.64	Thylakoid membrane of chloroplast	ATP synthesis, ion transport	Translocase	↓	↑	↑
Carbohydrate Metabolism									
8	Fructose bisphosphate aldolase	6.49	40.95	Chloroplast, cytoplasm	Glycolysis	Carbohydrate lysis	=	↓	=
13<br>16	Glyceraldehyde-3-phosphate dehydrogenase	9.05<br>5.91	43.20<br>36.07	Cytoplasm	Glycolysis	Oxidoreductase	=	↓<br>↓	↓<br>↑
19	Malate dehydrogenase	5.74	34.77	Cytoplasm, mitochondria	Citric acid cycle	Oxidoreductase	↑	↓	↑
20	Phosphoglycerate kinase	6.93	48.48	Cytoplasm	Glycolysis	ATP binding, phosphoglycerate kinase activity	↑	↓	↑
36	NADP- dependent oxidoreductase	7.57	33.31	Cytoplasm	Metabolism of small molecules and organic substances	Oxidoreductase	↑	=	↑

Table S4. (continued)

Defense and Stress Response									
21	Heat shock protein 70B	5.15	72.08	Cytoplasm, endosomes	Response to stress, protein folding	ATP binding; chaperone activity (binding of unfolded proteins)	=	↓	=
31	Antifreeze	9.68	27.43	Cytoplasm	Response to stress, ice recrystallization inhibition, pathogen defense	Binding to ice particles, thermal hysteresis	↑	↑	↑
32	Superoxide dismutase	8.63	26.06	Cytoplasm, chloroplast, mitochondrion, peroxisomes	Response to oxidative stress	Metal binding, oxidoreductase, superoxide dismutase	=	=	↑
35	Lactate dehydrogenase	4.98	24.34	Cytoplasm	Response to stress	Glyoxalase activity between lactate and methylglyoxal	↑	=	↑
Signal Transduction									
4<br>9<br>17<br>18	Calcium-dependent protein kinase and calmodulin	8.78<br>7.71<br>7.71<br>7.71	40.69<br>38.49<br>38.49<br>38.49	Cytoplasm	Signal transduction	ATP binding, protein serine/threonine kinase activity	↓<br>↓<br>=<br>=	↓<br>↓<br>↓<br>↓	=<br>↓<br>=<br>=
23	Rhodanase	4.63	39.45	Mitochondria	Response to Ca^{2+} stimulus	Sulphur transport	↓	=	↑
30	Voltage-dependent ion channel	8.51	28.73	Outer membrane of mitochondria	Ions, signals, and calcium transport	Ion channel	↑	↓	=
Transcription and Translation Processes									

Table S4. (continued)

7	Translational GTPase	8.73	50.82	Cytoplasm	Protein biosynthesis	GTP binding for aminoacyl-tRNA docking to the ribosome	=	↓	↓
37	Elongation factor Tu	5.36	44.9	Mitochondria, chloroplast	Protein synthesis and translation	GTP binding, GTPase	=	↓	=
2		5.36	44.9					↓	
33	RRM domain-containing protein	5.07	25.57	Nucleus, ribosomes	Translation, mRNA processing	RNA binding	↑	=	↑
Storage protein									
14	Cupin type 1	9.26	21.9	Cytoplasm	Storage protein	Nutrient storage	↑	=	↑
Mechanical Support of the Cell									
24	Actin	5.3	41.77	Cytoskeleton	Maintenance of cellular structures	Hydrolase	=	↑	↑

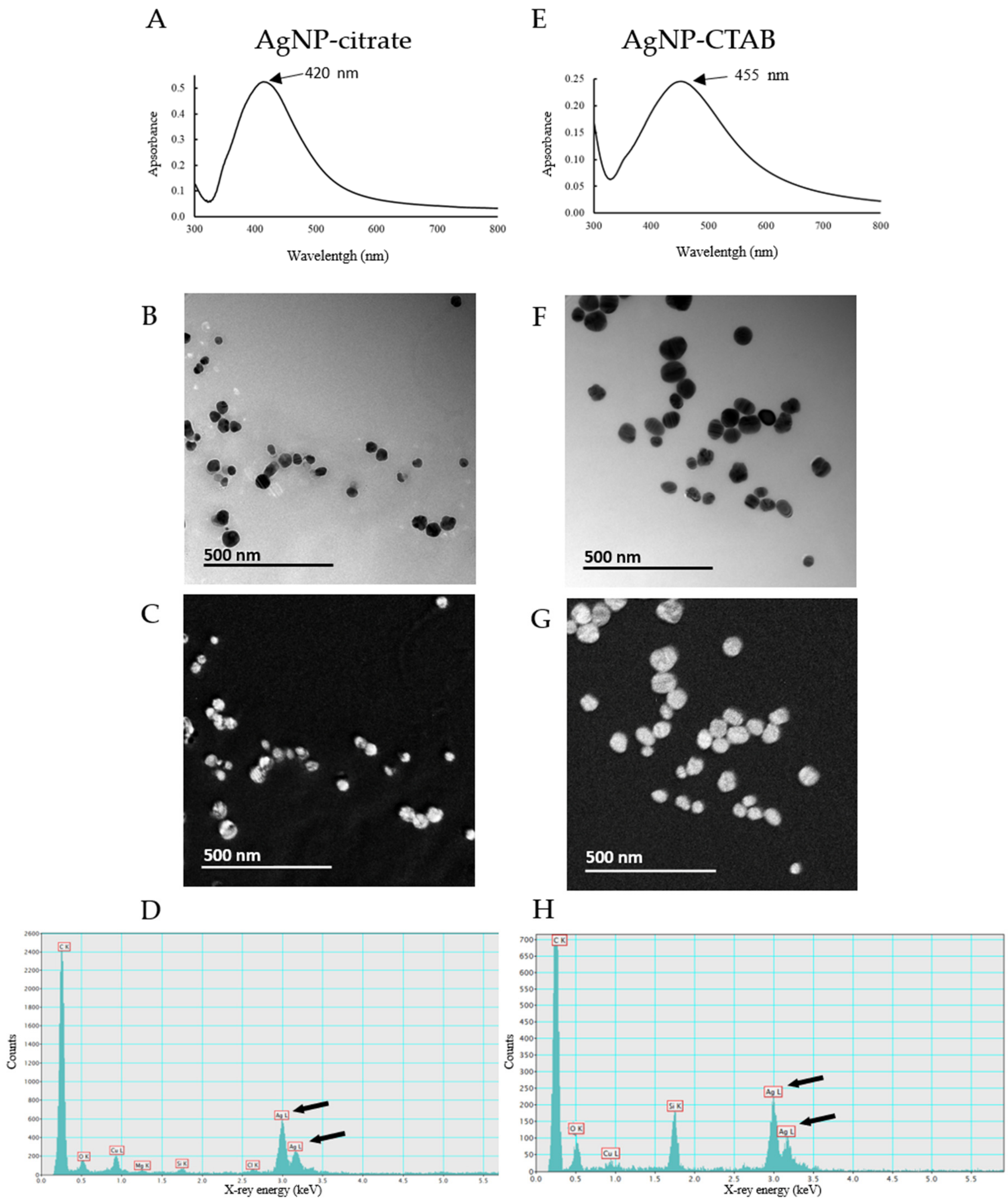

Figure S1: UV/Vis absorption spectra show absorbance peaks marked by arrows (A and E), while AgNP-citrate (B–D) and AgNP-CTAB (F–H) particles are visualized using transmission electron microscopy (TEM). Panels B and F display particles in bright field; C and G present silver element distribution maps, and D and H show EDX spectra, with arrows indicating the characteristic X-ray peaks of silver. Four independent replicates were analyzed for each stock suspension (n = 4). Scale bar represents 500 nm.
