# Supplementary material for: Multi-Parameter Analysis of Photosynthetic and Molecular Responses in Chlorella vulgaris Exposed to Silver Nanoparticles and Ions
Source: Toxics. 2025 Jul 26;13(8):627. doi: 10.3390/toxics13080627 (PMC12389777; doi:10.3390/toxics13080627)
Supplement: Supplementary file 1 [file toxics-13-00627-s001.zip › Table S1_final_proofread.pdf]

Table S1. Parameters of peptide analysis by Agilent 1290 Infinity II ultra-high-performance liquid chromatography (UHPLC).

Analytical column	*Agilent AdvanceBio Peptide Map,* 1.0 × 150 mm	
Column temperature	60 °C	
Autosampler temperature	10 °C	
Flow	0.1 mL min^{-1}	
Injection volume	8 μL	
Mobile phase A	0.1% FA / H_2O	
Mobile phase B	0.1% FA / 95% ACN	
Gradient	Time (min)	%B
	0	3
	10	40
	14	90
	16	90
	18	3
	21	3
