# Supplementary material for: Multi-Parameter Analysis of Photosynthetic and Molecular Responses in Chlorella vulgaris Exposed to Silver Nanoparticles and Ions
Source: Toxics. 2025 Jul 26;13(8):627. doi: 10.3390/toxics13080627 (PMC12389777; doi:10.3390/toxics13080627)
Supplement: Supplementary file 1 [file toxics-13-00627-s001.zip › Table S2_final_proofread.pdf]

Table S2. Parameters of peptide analysis by Agilent 6545 Q-TOF mass spectrometer (MS).

Ionization method	ESI with positive ionization
Gas temperature (N_2)	325 °C
Gas flow	13 L min $^{-1}$
Nebulizer	35 psi
Sheath gas temperature	275 °C
Sheath gas flow	12 L min $^{-1}$
Capillary voltage	4000 V
Nozzle voltage	0V
Fragmentor voltage	175 V
Skimmer voltage	65 V
Octopole RF Vpp	750 V
MS recording range (m/z)	100 - 2000
MS acquisition speed (spectra/s)	3
Auto MS/MS recording range (m/z)	100 - 2000
MS/MS acquisition speed (spectra/s)	2
Insulation width	Medium (~4 amu)
Maximum number of precursors per cycle	10
Precursor sorting	First by charge, then by intensity
Collision energy	3.1 × (*m*/*z*)/100+1 for charge 2^+<br>3.6 × (*m*/*z*)/100-4.8 for charge 3^+
MS/MS threshold (absolute number)	3000
Threshold for MS/MS (rel.) (%)	0.001
Dynamic shutdown	Recording 2 spectra, and switching off for a period of 0.05 min
MS/MS accumulation time frame	25,000 " *counts* " per spectrum
Reference ions during analysis	*m*/*z* 121.050873 (purines)<br>*m*/*z* 922.009798 (HP-921)
