# Supplementary material for: Multi-Parameter Analysis of Photosynthetic and Molecular Responses in Chlorella vulgaris Exposed to Silver Nanoparticles and Ions
Source: Toxics. 2025 Jul 26;13(8):627. doi: 10.3390/toxics13080627 (PMC12389777; doi:10.3390/toxics13080627)
Supplement: Supplementary file 1 [file toxics-13-00627-s001.zip › Table S3_final_proofread.pdf]

Table S3. The physicochemical properties of AgNP-citrate and AgNP-CTAB in their stock suspensions include the hydrodynamic diameter (d_H) derived from volume-based size distribution, ζ potential, total silver concentration, and the percentage of silver present as ions (Ag^+).

Property	AgNP-citrate	AgNP-CTAB
Hydrodynamic diameter (d_H), nm	43.1 ± 0.8	80.1 ± 1.5
ζ potential, mV	-45.62 ± 2.68	39.89 ± 1.79
Concentration, mg L^{-1}	99.8	89.2
Ag^+, %	0.5	0.5
