# Supplementary figures and images for: Multi-Parameter Analysis of Photosynthetic and Molecular Responses in Chlorella vulgaris Exposed to Silver Nanoparticles and Ions
Source: Toxics. 2025 Jul 26;13(8):627. doi: 10.3390/toxics13080627 (PMC12389777; doi:10.3390/toxics13080627)

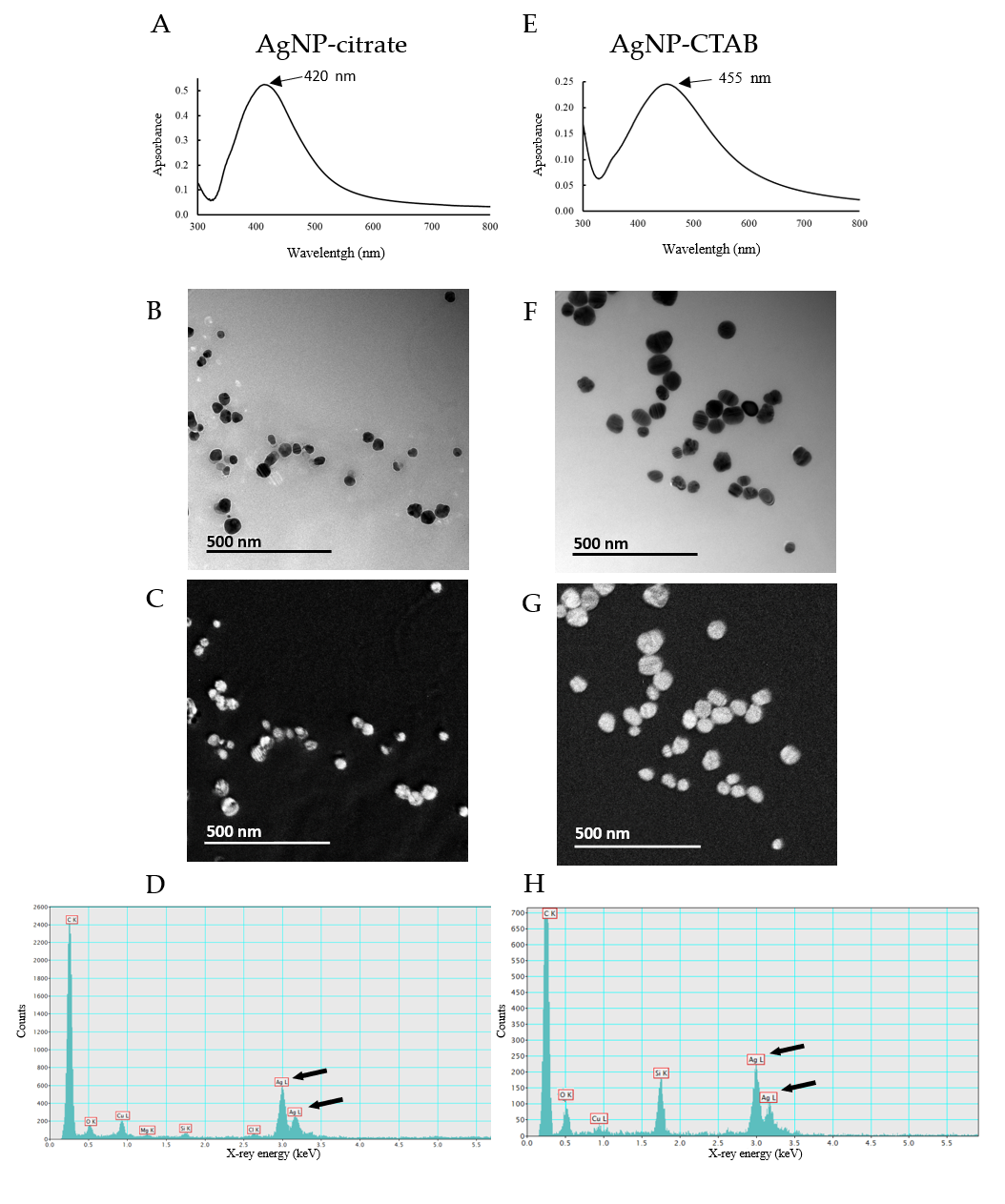

Supplement: Supplementary file 1 [file toxics-13-00627-s001.zip › Figure S1_final_proofread.png]
